# Ethnobotany of medicinal plants in Ada’a District, East Shewa Zone of Oromia Regional State, Ethiopia

**DOI:** 10.1186/s13002-015-0014-6

**Published:** 2015-04-02

**Authors:** Alemayehu Kefalew, Zemede Asfaw, Ensermu Kelbessa

**Affiliations:** Department of Plant Biology and Biodiversity Management, College of Natural Sciences, Addis Ababa University, 3434, Addis Ababa, Ethiopia

**Keywords:** Ada’a District, Ethnomedicinal knowledge, Scoring and ranking, Traditional medicinal plants

## Abstract

**Background:**

An ethnobotanical study of medicinal plants was conducted in Ada’a District, Eastern Shewa Zone of Oromia Regional State of Ethiopia. The objective of the study was to identify and document medicinal plants and the associated ethnobotanical/ethnomedicinal knowledge of the local people.

**Methods:**

Relevant ethnobotanical data focused on medicinal plants and traditional herbal medicines were collected using guided field walk, semi-structured interview and direct field observation. Informant consensus method and group discussion were conducted for crosschecking and verification of the information. Both descriptive statistics and quantitative ethnobotanical methods were used for data analysis.

**Results:**

We documented 131 species distributed in 109 genera and 54 families based on local claims of medicinal values. Patients who are using traditional drugs and herbalists collect most of these plants from the wild. The leading plant families that encompass large medicinal species were the Lamiaceae (14 species) followed by Asteraceae (13) and Solanaceae (7).

**Conclusion:**

The study reported the existence of a number of medicinal plants, an indication for the presence of plant-based traditional medicinal knowledge transfer that survived through generations. Informants asserted that wild growing medicinal plants are under threat due to increased use pressure coupled with unsuitable harvesting that frequently targets roots and barks for remedy preparations. This calls for urgent and collaborative actions to keep the balance between medicinal plants availability in the wild state and their utilization by the community. Furthermore, the study attempted to prioritize the most efficacious medicinal plants as perceived by the local people for possible pharmacological testing

## Background

Ethnomedicine studies the traditional medical practice and is concerned with the cultural investigation of health, disease and illness and also addresses the healthcare seeking process and healing practices [1-5]. Traditional methods of healing have been beneficial in many countries with or without access to conventional allopathic medicine. Ethiopia, is extremely heterogeneous ecologically [6-8] being a land of topographic diversities [9] and home of multiple ethnolinguistic groups [10]. Moreover, it is known to be a land for the origin of both human kind [11] and plants including crop species [12]. Thus, no wonder that it has diverse indigenous cultures that are carried over from past generations [9,13]. One aspect of this indigenous knowledge that began since time immemorial and applied for treating various ailments of human beings and domestic animals is herbal medicine. In agreement with this observation, various magico-religious literature sources [14,15] have noted that Ethiopia has a long history of applying traditional medicines for combating various ailments of humans and livestock.

In Ethiopia, traditional medicine is an integral part of the local culture and is a major public health system [16,17]. In addition to its deep cultural rooting, one reason for this is inaccessibility of modern healthcare services. According to the Health Sector Development Program (HSDP) of the Ethiopian Ministry of Health, the national standard is given as one hospital for 100, 000 people; one health centre is for 25, 000 people and one health post is for 5,000 people. On top of this, the country faces shortage of allopathic health professionals and the ratio of one doctor is for 10, 000 people; one nurse is for 5,000 people, one health extension worker is for 2,500 people [18]. Thus Traditional medicine (TM) is an important means of primary healthcare for achieving the goal, ‘Health for all’. The various literature sources available also support this fact where more than 70% of human and 90% of livestock population in Ethiopia depend on traditional medicine [19-23]. This tells us that medicinal plants and knowledge of their use provide a vital contribution to human and livestock healthcare throughout Ethiopia.

Similar to elsewhere in Ethiopia, people living in Ada’a District have also traditional practices which they put into effect for generations to take care of themselves and their livestock health. On the other hand, the area has been losing its indigenous flora due to the on-going human and natural causes [24]; and this loss of flora is associated with the missing of important indigenous knowledge on the plants and the traditional medical system. In strengthening this thought, several authors [25,26] noted that intense utilization of forests endangers the revival of the natural vegetation, in general and medicinal plants in particular; thus studying the ethnomedicine (herbal medicine in particular) could be considered as one of the conservation efforts in addition to other benefits in the context of driving many useful experiences for new scientific findings/innovations.

Though we have these facts, literature survey on the ethnobotanical investigation reveals that there is no previously conducted documentation work in any place in the District. Hence, there is a clear need to conduct ethnobotanical study of medicinal plants in the area, to look into and compile relevant information and to document them before the plants become too scarce to capture the knowledge of the indigenous people; and hence this study was initiated.

## Methods

### Study area

**Figure 1:**
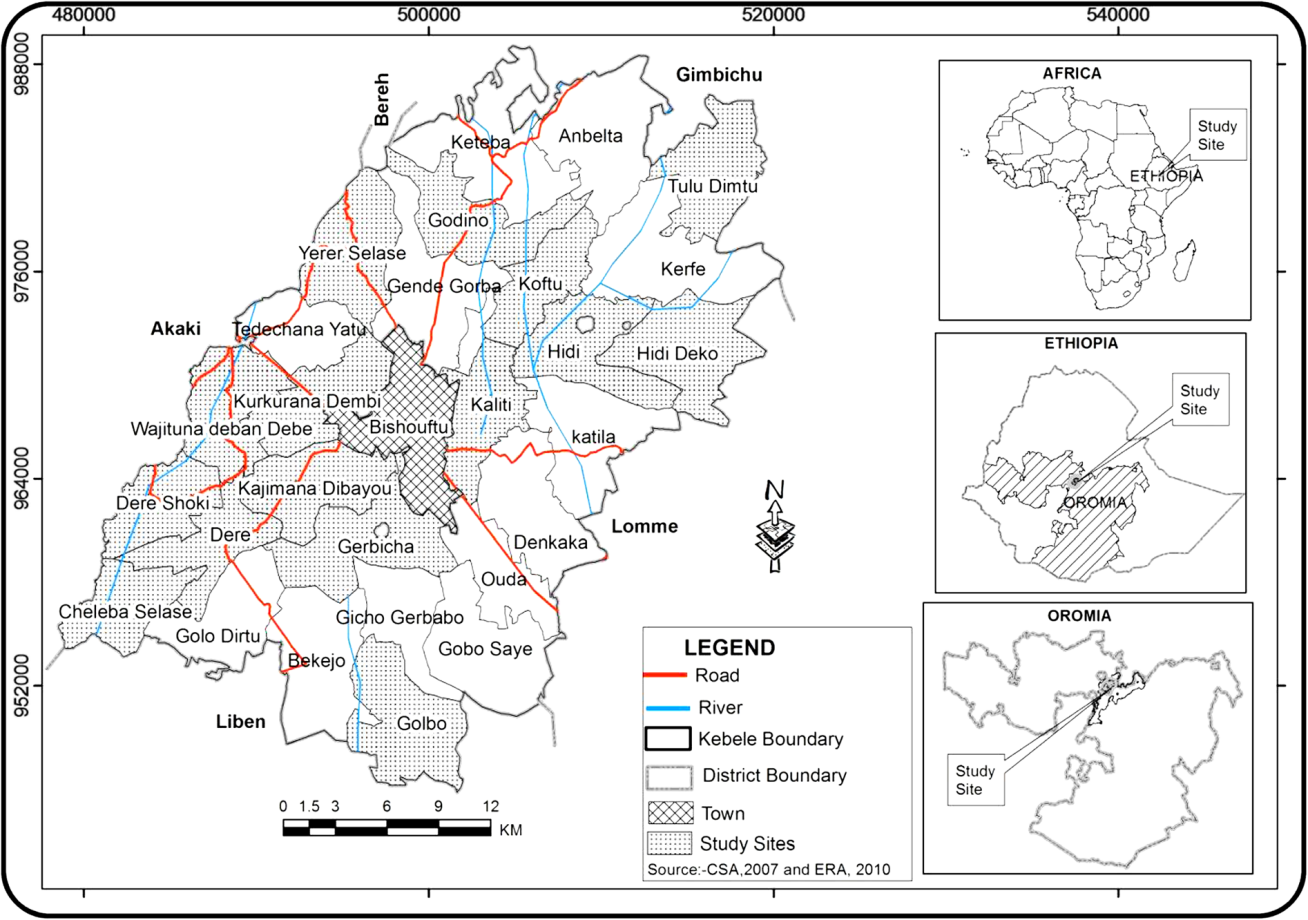
**Map showing the location of study sites in Ada’a District.**

This study was conducted is Ada’a District which has a total area of 96, 680 hectares. The area is one of the thirteen Districts in East Shewa Zone of Oromia Regional State of Ethiopia (Figure 1) and is located in the Great Rift Valley [24]. The relative location of the District is at about 45 km southeast of Addis Ababa, capital of the country. The District’s geographical location as indicated by the Ethiopian Mapping Authority (EMA) satellite image is 08°44′E latitude and 38°58′N longitude with an altitudinal range of 1540–3100 m a. s. l. [24,27].

### Sampling design

Out of the 27 Kebeles (the smallest administrative units) in the District, 15 (Chelebaselase, Dere, Dereshoki, Gerbicha, Godino, Golbo, Hidi, Hidideko, Kajimanadibayou, Kality, Koftu, Kurkuranadembi, Tuludimtu, Wajitunadebandebe, Yererselase) (55. 6%) were used as sampling kebeles for data collection. The selection of the 15 kebeles was made by purposive sampling based on the availability of traditional healers identified with the assistance of local authorities and elders. Within each kebele localities were identified based on eight habitat criteria (Forest lands, woodlands, grazing lands, fallow land, grasslands, wetlands, cultivated farm land, and home gardens). This procedure gave 140 total localities from which 101 sampling units were selected by taking one locality for each habitat type in each kebele using the lottery method (Table 1). This stratification procedure gave 8 forest land localities, 15 woodlands, 7 wetlands, 11 grasslands, 15 croplands, 15 grazing lands, 15 fallow lands and 15 home gardens that were employed for the collection of relevant data. The selection of localities based on stratification by habitat type was chosen as it is the best representative sample for capturing the medicinal plant and ethnomedicinal knowledge in the District. It is noted that not each kebele was represented by each of the habitat types.

**Table 1:** **Total number of localities and number of sampling units in Ada’a District**

		**Localities and sampling units for each habitas corresponding to each kebel**																
**S/N**	**Kebeles**	**Forest land**		**Woodland**		**Wetland**		**Grass land**		**Crop land**		**Grazing land**		**Fallow land**		**Home gardens**		**Total number of TNL, CNL per kebele**
		**TNL**	**CNL**	**TNL**	**CNL**	**TNL**	**CNL**	**TNL**	**CNL**	**TNL**	**CNL**	**TNL**	**CNL**	**TNL**	**CNL**	**TNL**	**CNL**	
1	Tulu Dimtu	1	1	2	1	1	1	NR	NR	2	1	1	1	1	1	2	1	10,7
2	Godino	1	1	2	1	NR	NR	1	1	2	1	1	1	1	1	1	1	9,7
3	Yererselase	2	1	2	1	1	1	1	1	1	1	2	1	1	1	1	1	12,8
4	Koftu	NR	NR	3	1	1	1	NR	NR	2	1	1	1	1	1	3	1	11,6
5	Hidideko	1	1	2	1	NR	NR	2	1	1	1	1	1	1	1	2	1	10,7
6	Hidi	NR	NR	2	1	NR	NR	1	1	2	1	1	1	1	1	2	1	10,6
7	Kality	1	1	2	1	1	1	1	1	2	1	1	1	1	1	1	1	10,8
8	Kurkuranade	1	1	2	1	1	1	1	1	1	1	2	1	1	1	3	1	12,8
9	Dereshoki	NR	NR	2	1	1	1	NR	NR	2	1	2	1	1	1	1	1	9,6
10	Dere	NR	NR	2	1	NR	NR	2	1	1	1	1	1	1	1	1	1	8,6
11	Kajimanadidayou	1	1	3	1	NR	NR	NR	NR	1	1	1	1	1	1	1	1	8,6
12	Gerbicha	NR	NR	3	1	1	1	1	1	1	1	1	1	1	1	1	1	9,7
13	Wajitunadedandede	NR	NR	2	1	NR	NR	1	1	3	1	1	1	2	1	2	1	11,6
14	Chelebaselase	NR	NR	1	1	NR	NR	1	1	2	1	1	1	1	1	1	1	7,6
15	Golbo	1	1	2	1	NR	NR	1	1	1	1	1	1	1	1	3	1	10,7
Total number of Localities		9	-	32	-	7	-	13	-	23	-	15	-	16	-	25	-	140
Total number of sampling units		-	8	-	15	-	7	-	11	-	15	-	15	-	15	-	15	101

N.B: TNL-Total number of localities, CNL-Chosen number of locality, NR-Not represented.

### Informant selection

Informants whose age ranged from 18 to 85 were chosen both purposively and randomly from among those born or have lived there for most of their lives. A total of 105 informants (69 males and 36 females) were used from 15 kebeles (7 informants per kebele). Sixty of the total informants (4 per kebele) were randomly selected. This was done in various ways. Some of them were chosen by tossing a coin and using him/her as informant whenever head of the coin was up if s/he volunteered to participate. Some others were chosen accidentally during random walk made to houses in the selected areas. The other 45 of the total informants (3 per kebele) were local experts (key informants) that were selected purposively based on recommendations from the local people, local authorities and development agents (DAs) at each study site.

### Ethnobotanical data collection

For ethical reasons, data were collected in the presence of local administrators and with the consent of each informant for the publication of the research and any accompanying images. Materials used for data collection were mainly plant press for specimen collection, Garmin GPS 72 int’l, digital photo camera and Walkman tape recorder. Ethnobotanical data collection was undertaken during two rounds of field visits during September 2009 to June 2010. The methods employed in the data collection were group discussion, semi-structured interviews, field observation, market survey, scoring and ranking. A checklist of semi-structured questions consisting of issues was prepared in advance. The interviews were done on and around this checklist and some issues were raised depending on responses of informants. During the course of the study, each informant was visited 2–3 times in order to validate the reliability of the ethnobotanical information. The visits were done without planned appointments with the informants. Consequently, the responses of an informant that were not in harmony with each other were considered vague and discarded from the analysis.

Field walk with guides and traditional healer(s) were made during the feasibility study. Group discussions, which were employed in each kebele, were used for cross-checking and verifying the information that has been gathered from individuals by semi-structured interview. The discussions were made with key informants, other traditional healers and the local people sometimes altogether or alone in their categories during field study; and that information was recorded using tape-recorder.

### Plant identification

Voucher specimens of medicinal plants that were reliably reported two times or more during informant visits were collected with the exception of some very common cultivated plants which were identified in the field. These specimens were gathered from the wild, from home gardens and crop fields and preliminary identification of these specimens was made in the field; and they were pressed and taken to the National Herbarium (ETH) of Addis Ababa University (AAU) where they were dried, deep frozen and identified. The identifications were done first using keys of published volumes of the relevant Flora of Ethiopia and Eritrea, and later supported with identification by comparisons with already authenticated specimen in the Herbarium. At last, they were confirmed with the help of taxonomic experts in Addis Ababa University (AAU); and all these voucher specimens were deposited at the National Herbarium.

### Data analysis

Both qualitative and quantitative analytical tools were used for data analysis following the approaches of Martin [1] and Cotton [3]. Percentage frequency method of data analysis was employed to summarize some of the descriptive ethnobotanical data obtained from the interviews on reported medicinal plants and associated knowledge. Microsoft Excel spread sheet was employed for organizing some ethnobotanical data. Preference ranking was performed to analyse most popular and preferred medicinal plants (MPs), at least in the context of the people who used them against blackleg, which was one of the most frequently reported livestock disease in the area. Direct matrix ranking was done to rank up medicinal plants reported frequently with various ethnobotanical roles.

Informant consensus factor (ICF) was used to find out most trusted healing plants for those disease categories that are claimed to be more common in the district following the approach of Heinrich and co-workers [28] by using the following formula:

$$ ICF=\frac{N_{UC}-{N}_S}{N_{UC}-1} where\;{N}_{UC}= number\; of\; use\; citation\;(report)\; in\; disease\; category; $$

$$ {N}_S= number\; of\; species\; used\;for\; each\; citations\;(report) $$

Other researchers [29] have also shown that this is a good means of assessing the agreements of informants on the common ailment categories, and thus we used it to test the consensus of the people in the District on curing the disease categories for which the plants were claimed to be effective.

Fidelity level/Species consensus has also been employed to rate the comparative curative capacity of reported traditional medicinal plants (TMP); and it was calculated by applying the formula: $FL=\left(\frac{S_f}{T_f}\right)\times 100\; where\;{S}_f\; refers\;to\; frequency\; of\; citations\;for\;a\; specific\; ailment;$

*T*_*f*_*refers to total number of citations of that species*. In this analysis, the consensus report of a species for treating a particular disease is seen with the report of that species for treating any given disease in the district [30,31].

## Results

### Diversity of medicinal plants (MPs) in the study area

**Figure 2:**
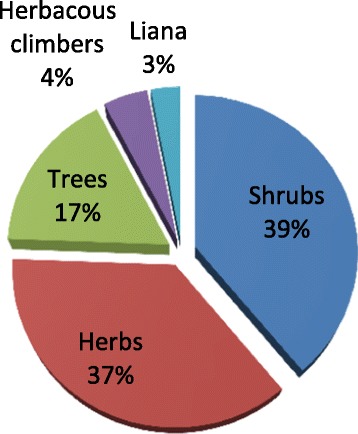
**Growth form of medicinal plants in Ada’a District.**

A total of 131species of MPs were gathered that were grouped under 109 genera and 53 families (Table 2). Of these plants, shrubs took the highest proportion (39%) whereas lianas took the least proportion (Figure 2).

**Table 2:** **Taxonomic diversity of medicinal plants in Ada’a District**

**Families**	**No. of genera**	**% of genera**	**No. of species**	**% of species**
Lamiaceae	12	11.0	14	10.6
Asteraceae	11	10.1	12	9.2
Solanaceae	5	4.5	7	5.3
Euphorbiaceae	3	2.7	5	3.8
Fabaceae	3	2.7	5	3.8
Apiaceae	4	3.6	4	3.1
Other 48 families	71	65.2	84	64.1

### Plant parts used for remedy preparation

**Figure 3:**
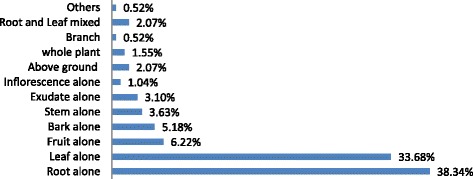
**Plant parts used for remedy preparation in Ada’a District.**

Various plant parts were reported for remedial preparation in the District (Figure 3). Roots were found to be the most familiar plant part for remedy preparations (74 species, 38.34%) followed by Leaf (65 species, 33.68%), fruits (12 species, 6.22%) and many other parts (42 species, 32.06%).

### Treated ailments and frequently reported medicinal plants

The MPs reported from the study area were used to treat both human and animal ailments. Among the documented MPs, 85 (64%) were those claimed to be used to treat human diseases (Table 3), 19 (15%) to treat livestock ailments (Table 4) and about 27 (21%) to treat both livestock and human diseases (Table 5). Among the medicinal plants, *Allium sativum*, *Rubia cordifolia*, and *Ruta chalepensis* were claimed to be the most frequently used medicinal plants as evidenced by the higher number of informant citations (Table 3, Table 4, and Table 5). Details on the mode of preparations and applications of remedies are given in the appendix (Appendix 1).

**Table 3:** **List of traditional medicinal plant (MP) species used to treat human ailments in Ada’a District**

**S/N**	**Scientific name**	**Family**	**Local name (Afan Oromo/Amaric)**	**Coll. from**	**Ha.**	**UT**	**TNC**	**Alt. Range**	**Coll. No**
1	Acacia seyal Del.	Fabaceae	Wachoo/Wachu	W/CL	T	Hu	3	1500-2200	AK 186
2	*Achyranthes aspera* L.	Amaranthaceae	Derguu/Etse-tekeze	W	H	Hu	20	1600-2500	AK 003
3	*Acmella caulirhiza* Del.	Asteraceae	Guticha	W	H	Hu	7	2100-2500	AK 150
4	*Ageratum houstanianum* Mill	Asteraceae	Q/Merzi/Yemerz Medanit	W	H	Hu	5	1800-2500	AK 265
5	*Ajuga integrifolia* Buch. Ham.	Lamiaceae	Harmmaguusa/Aqorarache	W/CL	H	Hu	21	1900-2600	AK 004
6	*Allium cepa* L.	Alliaceae	Shunkurtiidiimaa/Keyshinkurt	HG	H	Hu	8		AK 185
7	*Allium sativum* L.	Alliaceae	Qullubbi adii/Nech shinkurt	HG	H	Hu	32		AK 005
8	*Alternanthera pungens* Kunth.	Amaranthaceae	*****	W/CL	H	Hu	3	1400-1900	AK 228
9	*Artemisia absinthium* L.	Asteraceae	Harritta/Aritii	HG	H	Hu	8		AK 184
10	*Artemisia abyssinica* Schtz. Bip. ex Rich	Asteraceae	Tiroo/Chikugne	W	H	Hu	27	2400-2700	AK 144
11	*Asparagus africanus* Lam.	Asparagaceae	Seriiti/Seriti	W	S	Hu	7	1500-2700	AK 064
12	*Asparagus racemosus* Wild.	Asparagaceae	Seriiti/Seriti	W	S	Hu	9	1600-2700	AK 227
13	*Asplenium monanthes* L.	Aspleniaceae	*****	W	H(F)	Hu	2	1600-2600	AK 009
14	*Bidens pilosa* L.	Asteraceae	Chogogitii/Chogogit	W/CL	H	Hu	5	1500-2500	AK 066
15	*Caparis tomentosa* Lam.	Capparidaceae	Goora/Gumero	W	CL	Hu	9	1600-2100	AK 243
16	*Capsicum annuum* L.	Solanaceae	Qaara/Qariya	HG	H	Hu	5		AK 012
17	*Carissa spinarum* (Vahl.) Forssk. ex Endl.	Apocynaceae	Agamsa/Agam	W	S	Hu	5	1650-2600	AK 180
18	*Catha edulis* (Vahl.) Forssk. ex Endl.	Celastraceae	Caatii/Chat	HG	T	Hu	3		AK 223
19	*Centella asiatica* (L.) Urban.	Apiaceae	*****	W	H	Hu	4	1800-2400	AK 179
20	*Citrus* aurantifolium (L.) Burn. f.	Rutaceae	Loomii/Lomi	HG	S	Hu	5		AK 222
21	*Clausena anisata* (Wild.) Benth.	Rutaceae	Ulumaa/Limich	W	S	Hu	7	2000-2400	AK 140
22	*Clerodendrum myricoides* (Hochst) Vatke	Lamiaceae	Maraasisaa/misirich	W	S	Hu	4	2000-2500	AK 221
23	*Colocasia esculenta* (L.) Schott	Araceae	Godaree/Godore	HG	H	Hu	4		AK 067
24	*Croton macrostachyus* Del.	Euphorbiaceae	Bakaniisaa/Bisana	W	T	Hu	11	1600-2500	AK 017
25	*Cucumis dipsaceus* Ehrenb.	Cucurbitaceae	Buqee seexanaa/Yesetan kil	HG	CL	Hu	8		AK 068
26	*Cucumis ficifolius* A. Rich.	Cucurbitaceae	Holoo/Yemidir enbuay	W	CL	Hu	14	1600-2000	AK 219
27	*Cyathula cylindrica* Moq.	Amaranthaceae	Derguu/Yemogne Fikir	W	H	Hu	4	1700-2600	AK 137
28	*Cymbopogon citratus*	Poaceae	Xajisaara/Tej sar	HG	H	Hu	4		AK 069
29	*Datura stramonium* L.	Solanaceae	Atsefaris/Astenagir	W	H	Hu	5	2000-2500	AK 217
30	*Dombeya torrida*(J. F. Gmel) Bamps	Sterculiaceae	Daanisa/Wolkefa	W	T	Hu	3	2500-2700	AK 175
31	*Dregea schimperi*(Decne.) Bullock	Asclepiadaceae	Hida/Yeregna missa	W	LI	Hu	4	1900-2400	AK 021
32	*Ekebergia capensis* Sparrm.	Meliaceae	Somboo/Sombo	W	T	Hu	6	2000-2700	AK 022
33	*Eleusine floccifolia* Forssk.	Poaceae	Coqorsa/Akerma	W/CL	H	Hu	7	2100-2500	AK 214
34	*Embelia schimperi* Vatke	Myrsinaceae	Hanquu/Enqoqo	W	S	Hu	8	1800-2700	AK 134
35	*Eucalyptus globulus* Labill.	Myrtaceae	Nech bahir zaf	HG	T	Hu	11		AK 212
36	*Euclea racemosa* subsp. *schimperi*	Ebenaceae	Me’essaa/Dedeho	W	S	Hu	5	1500-2600	AK 173
37	*Euphorbia abyssinica* J. F. Gmel.	Euphorbiaceae	Adamii/Kulkual	HG	T	Hu	6		AK 073
38	*Euphorbia ampliphylla*	Euphorbiaceae	Adamii/Kulkual	HG	T	Hu	6		AK 025
39	*Euphorbia tirucallii* L.	Euphorbiaceae	Cadaa/Kinchib	HG	S	Hu	3	1500-2000	AK 132
40	*Ferula communis* L.	Apiaceae	Dog	W	H	Hu	6	2500-2700	AK 074
41	*Foeniculum vulgare* Mill.	Apiaceae	Insilaalee/Ensilal	W	H	Hu	23	2000-2600	AK 075
42	*Grewia ferruginea* Hochst ex . A . Rich.	Tiliaceae	Dhoqonuu/Lenquata	W	S	Hu	4	1700-2300	AK 209
43	*Guizotia scabra*(Vis) Chiov.	Asteraceae	Adaa/Mech	W	H	Hu	2	1700-2400	AK 030
44	*Heteromorpha trifoliata* (Wendel.) Eckl. & Zeyh.	Apiaceae	Demehee/Yejib merkuze	W	S	Hu	5	2200-2500	AK 207
45	*Hypericum quartinianum* A. Rich.	Hypericaceae	Muke fonii	W	S	Hu	4	2000-2500	AK 034
46	*Impatiens ethiopica* Grey-Wilson	Balsaminaceae	Yehenshoshilaa zer	HG	H	Hu	2		AK 206
47	*Impatiens rothii* Hook. f.	Balsaminaceae	Buri/Gesherit	W	H	Hu	2	2400-2600	AK 080
48	*Impatiens tinctoria s*ubsp. *abyssinica*	Balsaminaceae	Ensosilla	W	S	Hu	2	1900-2400	AK 035
49	*Jasminum grandiflorum* L.	Oleaceae	Qamaxee/Tembelel	W	S	Hu	6	1700-2500	AK 235
50	*Juniperus procera* Endle	Cupressaceae	Gaatiraa/Yehabesha Tid	W/HG	T	Hu	4	2100-2800	AK 081
51	*Lagenaria siceraria* (Molina) Standl.	Cucurbitaceae	Buqqee/Kil	HG	H	Hu	3		AK 238
52	*Laggera tomentosa* (Sch. Bip. ex A. Rich.) Oliv. & Hiern	Asteraceae	Keskeso	W	H	Hu	3	1700-2500	AK 166
53	*Lantana camara* L.	Verbenaceae	Yewof kolo	HG	LI	Hu	2		AK 038
54	*Leucas martinicensis* (Jacq. ) R. Br.	Lamiaceae	Bokkoluu adii/Ras kimir	W	S	Hu	8	1900-2400	AK 039
55	*Lippia adoensis* Hochst. ex Walp.	Verbenaceae	Kusaayee/Kese	W/HG	S	Hu	3	1800-2800	AK 084
56	*Mentha spicata*	Lamiaceae	Nana	HG	S	Hu	4		AK 224
57	*Myrica salicifolia* A. Rich.	Myricaceae	Kataba/Shinet	W	T	Hu	4	2200-2800	AK 232
58	*Myrtus communis*	Myrtaceae	Adasii/Ades	M	S	Hu	6		AK 088
59	*Nicotiana tabacum* L.	Solanaceae	Tamboo/Tembaho	HG	H	Hu	3		AK 044
60	*Ocimum basilicum* L.	Lamiaceae	Besobilla	HG	H	Hu	5		AK 122
61	*Ocimum gratissimum* L.	Lamiaceae	Q/Michii/Mech medanit	W	H	Hu	15	1700-2500	AK 161
62	*Ocimum lamiifolium* Hochst. ex Benth.	Lamiaceae	Demakessie	W/HG	S	Hu	24	1600-2600	AK 045
63	*Olea europaea* subsp. *Cuspidata*	Oleaceae	Ejersa/Weyra	W/HG	T	Hu	4	1900-2600	AK 090
64	*Olinia rochetiana* A. Juss.	Oliniaceae	Dalecho	W	S	Hu	6	2200-2600	AK 245
65	*Osyris quadripartita* Decn.	Santalaceae	Waatoo/Qeret	W	S	Hu	3	1900-2500	AK 160
66	*Otostegia integrifolia* Benth.	Lamiaceae	Tungiitii/Tungit	W	S	Hu	12	1800-2500	AK 047
67	*Plantago major* L.	Plantaginaceae	Qorxobbii/Yekura wesife	W	H	Hu	6	2000-2500	AK 120
68	*Premna schimperi* Engl.	Lamiaceae	Urgessa/Chchoho	W	S	Hu	4	2000-2500	AK 051
69	*Prunus persica* (L.) Batsch	Rosaceae	Kokkii/Kok	HG	S	Hu	2		AK 119
70	*Pterolobium stellatum* (Forssk.) Brenan	Fabaceae	Harengeemmaa/Kontir	W	S	Hu	5	1800-2400	AK 098
71	*Ricinus communis* L.	Euphorbiaceae	Qoboo/Gulo	HG	T	Hu	6		AK 118
72	*Rosmarinus officinalis* L.	Lamiaceae	Siga metsebesha	HG	S	Hu	13		AK 055
73	*Rumex nervosus* Vahl	Polygonaceae	Dhangaggoo/Embuacho	W	H	Hu	5	1800-2600	AK 103
74	*Ruta chalepensis* L.	Rutaceae	Xeenaadama/Tsenadam	HG	H	Hu	29		AK 112
75	*Salix mucronata*	Salicaceae	Alaletu/Ahaya	W	T	Hu	6	1700-2500	AK 153
76	*Salvia nilotica* Jacq.	Lamiaceae	Hulegebe	W/CL	H	Hu	3	1600-2800	AK 104
77	*Schinus molle* L.	Anacardiaceae	Kundoberbere zaf	HG	T	Hu	3		AK 152
78	*Snowdenia polystachya* (Fresen.) Pig.	Poaceae	Muja	W	H	Hu	2	1700-2200	AK 114
79	*Solanum marginatum* Linn. f.	Solanaceae	Hiddii/Tileku Enbuay	W/CL	S	Hu	5	1900-2600	AK 107
80	*Thunbergia alata* Sims.	Acanthaceae	Hareg	W	CL	Hu	3	2200-2500	AK 256
81	*Thymus schimperi* Ronniger	Lamiaceae	Xoosanyii/Tosigne	W	S	Hu	8	2500-2800	AK 108
82	*Urtica simensis* Steudel	Urticaceae	Dobii/Sama	W	H	Hu	2	2200-2700	AK 057
83	*Verbena officinalis* L.	Verbenaceae	Atuch	W/CL	H	Hu	9	2000-2400	AK 109
84	*Vernonia amygdalina* Del.	Asteraceae	Ebicha/Grawa	HG	T	Hu	13		AK 230
85	*Withania somnifera* (L.) Dunal.	Solanaceae	Gizaawaa/Gizawa	W	S	Hu	6	2400-2600	AK 110
86	*Zehneria scabra* L.	Cucurbitaceae	Daaymii/Areg resa	W	CL	Hu	9	1900-2500	AK 197

**Table 4:** **List of traditional medicinal plant (MP) species used to treat livestock ailments in Ada’a District**

**S/N**	**Scientific Name**	**Family**	**Local Name (Oromifa/Amarigna)**	**Coll. from**	**Ha.**	**UT**	**TNC**	**Alt.Range**	**Coll. No**
1	*Acacia abyssinica* Hochst. ex Benth.	Fabaceae	Laaftoo/Girar	W/CL	T	An	3	1500-2500	AK 147
2	*Acacia albida* Del.	Fabaceae	Garbii/Gerbi	W/CL	T	An	5	1500-2200	AK 001
3	*Agave sisalana* Perrineex Engel.	Agavaceae	Qachaa/Qacha	W/HG	T	An	3	1500-2300	AK 062
4	*Bersama abyssinica* Fresen.	Melianthaceae	Loliichisa/Azamir	W	T	An	4	1700-2600	AK 242
5	*Buddlejia polystachya* Fresen.	Buddlejiaceae	Qawissa/Anfar	W/HG	T	An	12	2000-2500	AK 142
6	*Dodonaea angustifolia* L. f.	Sapindaceae	Etacha/Kitkita	W	S	An	3	1800-2450	AK 216
7	*Gamphocarpus abyssinicus* Decne.	Asclepiadaceae	Rebu Hunda	W	H	An	9	2300-2500	AK 028
8	*Hypericum revolutum* Vahl	Hypericaceae	Hindhee/Ameja	W	S	An	3	2000-2500	AK 226
9	*Malva venticillata* L.	Malvaceae	Liitii/Lit	W	H	An	2	2000-2700	AK 040
10	*Pentas schimperiana* (A. Rich.) Vatke	Rubiaceae	Dasie	W	S	An	11	2100-2600	AK 049
11	*Plantago lanceolata* L.	Plantaginaceae	Qorxobbii/Yehaheya Kote	W/CL	H	An	4	1900-2500	AK 195
12	*Protea gaguedi* J. F. Gmel.	Proteaceae	Dasie	W	S	An	11	1900-2200	AK 241
13	*Rhus retinorrhoea*	Anacardiaceae	Tilem	W	S	An	4	2000-2700	AK 155
14	*Rhus vulgaris* Meikle	Anacardiaceae	Dabobechaa/Kimmo	W	S	An	3	1900-2800	AK 100
15	*Rosa abyssinica* Lindley	Rosaceae	Gora/Kega	W/CL	S	An	7	2100-2700	AK 192
16	*Sida schimperiana* Hochst. ex A. Rich.	Malvaceae	Chefreg	W	H	An	4	2100-2400	AK 191
17	*Tagetes minuta* L.	Asteraceae	Tiro	W	S	An	4	1600-2300	AK 255
18	*Xanthium strumarium* L.	Asteraceae	Yemogne Fikir	W/CL	S	An	3	1700-2500	AK 187

Key: − Coll- Collected; Coll. No – Collection number; Ha-Habit; UT-Used to treat; Alt. Range-Range of Altitudes distribution in meters; W-Wild; CL-Cropland; HG- Home Garden; M-Market; F-Fern; Hu-Human; An-Animal; Bo-Both human and animal; *****- Local name not known; TNC:-Total number of citations

**Table 5:** **List of traditional medicinal plant (MP) species used to treat both human and livestock ailments in Ada’a District**

**S/N**	**Scientific name**	**Family**	**Local name (Oromifa/Amarigna)**	**Coll. from**	**Ha.**	**UT**	**TNC**	**Alt. Range**	**Coll. No**
1	*Aloe macrocarpa* Tod.	Aloaceae	Argiisa/Ret	W/CL	H	Bo	6	1850-2150	AK 145
2	*Brucea antidysenterica* J. F. Mill.	Simaroubaceae	Qumegno/Abalo	W	S	Bo	15	1900-2700	AK 182
3	*Calpurnia aurea* (Ait.) Benth.	Fabaceae	Ceekaa/Digita	W	S	Bo	6	1600-2750	AK 225
4	*Clematis simensis* Fresen.	Ranunculaceae	Fiitii/Enderifa	W	LI	Bo	12	1800-2700	AK 178
5	*Cyphostemma adenocaule*	Vitaceae	Melas golgul	W	CL	Bo	14	2000-2450	AK 060
6	*Ficus sur* Forssk.	Moraceae	Harbu/Sholla	W	T	Bo	5	1750-2200	AK 210
7	*Fuerstia africana* Th. Fries	Lamiaceae	Eje Admek	W	H	Bo	11	1600-2200	AK 083
8	*Hygrophila schulli* (Hamilt.) M. R. & S. M. Almeida	Acanthaceae	*****	W/CL	H	Bo	3	1900-2400	AK 079
9	*Inula confertiflora* A. Rich.	Asteraceae	Mognoree/Weynageft	W	S	Bo	5	2200-2600	AK 253
10	*Justicia schimperiana* (Hochst. ex Nees) T. Anders	Acanthaceae	Dhumuugaa/Sensel	HG	S	Bo	27		AK 167
11	*Kalanchoe petitiana* A. Rich	Crassulaceae	Bosoqee/Endahula	W	H	Bo	24	1900-2600	AK 257
12	*Leonotis raineriana* Vis.	Lamiaceae	Bokkoluu dimma/Ras kimir	W	S	Bo	25	2400-2700	AK 125
13	*Maesa lanceolata* Forssk.	Myrsinaceae	Abbayyii/Kelewa	W	S	Bo	5	2100-2800	AK 202
14	*Melia azedarach* L.	Meliaceae	*****	HG	S	Bo	9		AK163
15	*Myrsine africana* L.	Myrsinaceae	Qacama/Kechem	W	S	Bo	9	2200-2600	AK 043
16	*Pavetta abyssinica* Fresen.	Rubiaceae	Muke-buniti	W	S	Bo	5	2000-2500	AK 027
17	*Phytolacca dodecandra* L’ Herit	Phytolaccaceae	Handoode/Endod	W/HG	S	Bo	19	2000-2700	AK 095
18	*Prunus africana* (Hook. f. ) Kalms	Rosaceae	Hoomii/Tikur Enchet	W/HG	T	Bo	14	2100-2600	AK 097
19	*Rubia cordifolia* L.	Rubiaceae	Enchibir	W	H	Bo	31	1800-2600	AK 111
20	*Rubus steudneri* Schweinf.	Rosaceae	Agogota	W	H	Bo	4	2500-2800	AK 082
21	*Rumex abyssinicus* Jacq.	Polygonaceae	Meqmeqo	W	H	Bo	9	2000-2800	AK 154
22	*Rumex nepalensis* Spreng.	Polygonaceae	Shuultii/Tulet	W	H	Bo	19	1700-2600	AK 231
23	*Solanecio gigas* (Vatke.) C. Jeffrey	Asteraceae	Gommana osolee	HG	S	Bo	7		AK 115
24	*Solanum anguivi* Lam.	Solanaceae	HiddiWorabessa/ZerchEnbuay	W/CL	S	Bo	8	1600-2700	AK 247
25	*Solanum incanum* L.	Solanaceae	Hiddii/Yehabesha Embuay	W/CL	S	Bo	6	1500-2400	AK 151
26	*Stephania abyssinica* (Dillon ex A. Rich.) Walp.	Menispermaceae	Kalaala/Engochit	W	LI	Bo	26	2000-2800	AK 189
27	*Verbascum sinaiticum* Benth.	Scrophulariaceae	Guraa Haree/Yahaya joro	W/CL	H	Bo	6	1900-2600	AK 149

### Preparation of remedies

**Figure 4:**
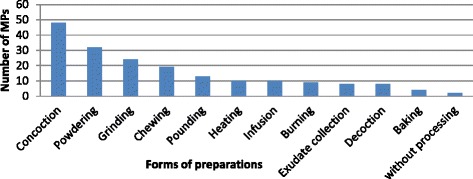
**Forms of MPs preparation in Ada’a District.**

Interview carried out with most of the healers of the study area revealed that herbal medications were prepared differently. They often have a preference of mixing two or more MPs so as to avoid or minimize side effect of the remedies. Most of the remedies were prepared in the form of concoction whereas one species (*Heteromorpha trifoliata*) was served as medicine without processing (Figure 4).

**Figure 5:**
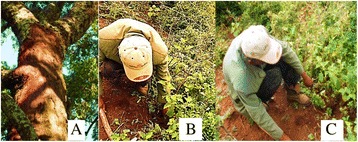
**Examples of bad harvesting system, A-debarking** ***Prunus africana*** **&** **B and C- uprooting of** ***Asparagus africana*** **for local remedial preparation in Ada’a District.**

Field observation revealed that healers use debarking, for instance *Prunus africana*, and uprooting, for example *Asparagus africanus*, as cases of unfavourable means of herbal collection for remedy preparation (Figure 5).

### Routes of application

The herbal drugs following preparation were reported to be administered in diverse routes (Table 6). The routes and method of applications in the study sites varies with the type of disease treated and the actual sites of the ailments.

**Table 6:** **Routes of application of remedies in Ada’a District**

**Routes of application**	**Number of MPs**	**% of MPs**
Oral	90	49.4
Dermal	70	38.4
Nasal	9	4.9
Auricular	6	3.2
Dental	4	2.1
Ocular	3	1.6
Nasal & auricular	2	1.1
Vaginal	1	0.5

### Conditions of medicine preparation

Most commonly, the local people asserted that they prefer the fresh plant part than the dried part for remedy preparation. Among the total MPs, 110 (57.89%) were used in the fresh form, 77 (40.53%) were used in the dried form. Only three plants (2%) were reported to be used in either form.

### Dosages and other related prescriptions

**Figure 6:**
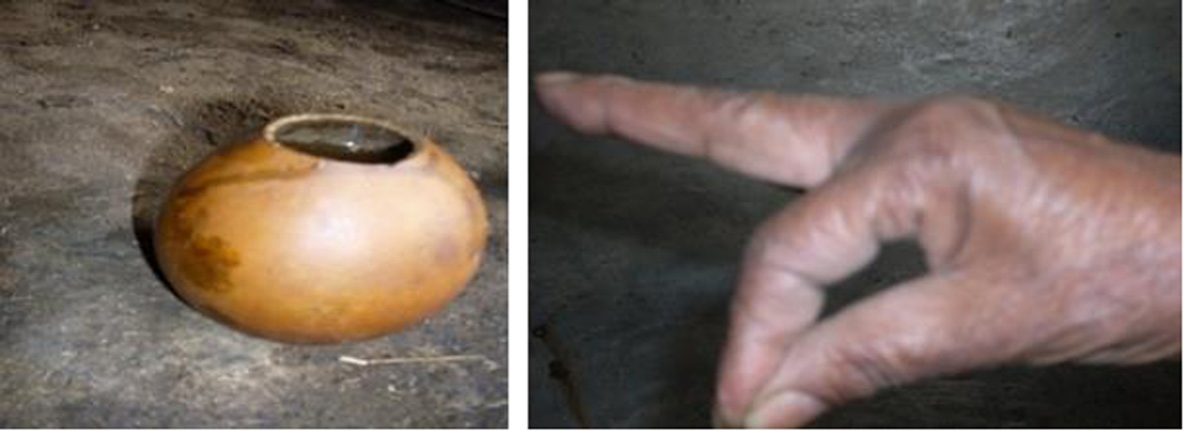
**Some ways for measuring doses of herbal medicine in the district (Left ANKOLA, Right BETAT).**

In this study, provisions of doses vary with age. Such cases were not noted for gender variations. Dose of decoction is measured in various ways (see Figure 6) including tea or coffee cups (small for children, and large-sized for youngsters), JOGE (known to be equivalent to a litre), glass for local liquor (locally called YEAREKE MELEKIYA), local alcoholic beverage cup (TELLA cup), and ANKOLA (a traditional cup made of dried fruit of *Lagenaria siceraria*). Powdered herbal materials were measured roughly on the palm described as BETAT (i. e., measured by holding the powders between the thump and next (index) finger). Visual observations during herbal preparations showed that palm sanitation of herbalists and container was not considered. Healers also prescribed a particular dose to be taken once, twice or three times per day after carrying out traditional physical examination like looking to patients palm or eye.

### Methods of applying treatments (Forms of therapy)

**Figure 7:**
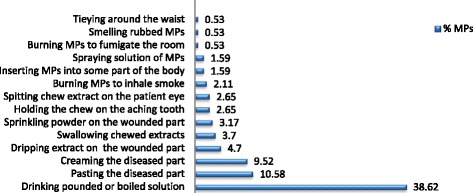
**Forms of applying traditional medication/thereapy in Ada’a District.**

The documented MPs were used to treat the reported ailments by applying in various ways (Figure 7). Most of the diseases that are inexplicable in the scientific world, like demon possession (*GANEN*), Evil eye (*BUDA*), depressions (*EJE SEB*) were found to be easily treated by healers; and medications were given in the name of WAAQAYOO/REBBY (a local term to mean the almighty God).

### Habitat and sources of medicinal plants

Among the whole MPs, 73 of them (55.72%) were collected from the wild, 28 (21.37%) from home gardens (HGs), 20 (15.26%) from both wild and cropland and 9 species (6.87%) from both wild and HGs and the remaining one species, *Myrtus communis*, was recorded from open market in the District. Those MPs that were obtained from both wild environment (such as forests, grassland, wetlands and so on) and croplands were found as naturally growing plants; and it was observed that they were open for any local people who need to use them. However, those MPs obtained in home gardens were primarily grown for the purpose of foods, as spices, for marketing, as fences, stimulants, and ornaments. In the open market the MPs were usually found in relation to seeds and fruits of spices and herbs.

### Most important medicinal plants

Ranking and scoring method is very helpful to compare and judge widely applicable MPs that have been assured through frequent citations. The results of simple preference ranking by key informants on five most cited MPs against blackleg are shown in Table 7 and direct matrix ranking of five most common multi-purpose MPs are given in Table 8.

**Table 7:** **Results of preference ranking for five MPs against blackleg (Scores in the table shows ranks given to medicinal plant based on their efficacy; thus 5 = most effective, 1 = least preferred)**

**Medicinal plant species For blackleg**	***Respondents (R1-R10)***											
	***R1***	***R2***	***R3***	***R4***	***R5***	***R6***	***R7***	***R8***	***R9***	***R10***	***Total***	***Rank***
*Cyphostemma adenocaule*	5	4	5	5	2	4	3	5	4	4	41	1^st^
*Verbascum sinaiticum*	4	3	2	2	1	1	2	4	3	5	27	4^th^
*Prunus africana*	5	5	4	3	3	5	5	3	2	3	38	2^nd^
*Rumex abyssinicus*	2	2	1	1	4	2	1	1	1	1	16	5^th^
*Stephania abyssinica*	3	4	3	4	5	3	4	2	5	2	35	3^rd^

**Table 8:** **Results of direct matrix ranking for five multi-purpose MPs in Ada’a District**

**Use category**								
**Plant species**	**Medicine**	**Food**	**Firewood**	**Charcoal**	**Construction**	**Share**	**Total**	**Rank**
*Juniperus procera*	*4*	*0*	*3*	*1*	*4*	*2*	*14*	*5* ^*th*^
*Acacia albida*	*4*	*0*	*3*	*4*	*3*	*1*	*15*	*4* ^*th*^
*Croton macrostachyus*	*5*	*0*	*2*	*3*	*3*	*4*	*16*	*3* ^*rd*^
*Olea europaea subsp*.	*4*	*2*	*2*	*3*	*3*	*4*	*18*	*1* ^*st*^
*Cuspidata*								
*Prunus africana*	*4*	*2*	*2*	*3*	*3*	*3*	*17*	*2* ^*nd*^

N. B: Number in the table shows average scores of four key informants given to each medicinal plants based on their multipurpose use categories.

### ICF and FL values

Calculation of ICF values showed the most effective medicinal plants against the common illness category of the District. Accordingly, seven disease categories that turned out ICF values greater than 0.78 were noted to be the more prevalent health problems in the District (Table 9). The highest plant use citation was recorded for the diseases categorized as gastro-intestinal disorders.

**Table 9:** **Results of Informants consensus factor (ICF) for more prevalent health problems of the District**

**More prevalent disease category**	**List of plant species used and number of citation in the bracket**	**Total no. of species**	**Total no. of citation**	**ICF**
Retained placenta	*Solanecio gigas*(7)	1	7	1.00
Skeleto-muscular disorder	*Ajuga integerifolia* (4), *Pterolobium stellatum* (5)	2	9	0.88
Febril illness & General malaise	*Allium sativum* (3), Croton *macrostachyus* (6), *Eucalyptus globulus* (7), *Fuerstia africana* (1), *Lantana camara* (2), *Leonotis raineriana* (1), *Leucas martinicensis* (8), *Myrtus communis* (3), *Ocimum gratissimum* (15), *Ocimum lamiifolium* (24), *Otostegia integrifolia* (12)	11	82	0.87
Circulatory disorder	*Allium cepa* (6), *Nicotiana tabacum* (3), *Thymus schimperi* (8)	3	17	0.87
Gastro-intestinal disorder	*Achyranthes aspera* (11), *Ajuga integerifolia* (8), *Allium sativum*(5), *Aloe macrocarpa* (3), *Artemisia abyssinica* (12), *Asparagus africanus* (7), *Asparagus racemosus* (9), *Capsicum annuum* (5), *Carissa spinarium* (3), *Citrus* x *limon* (5), *Clerodendrum myricoides* (4), *Croton macrostachyus* (5), *Cucumis ficifolius* (14), *Cyathula cylindrica* (4), *Embelia schimperi* (8), *Eucalyptus globulus* (4), *Foeniculum vulgare* (5), *Grewia ferruginea* (4), *Lippia adoensis* (4), *Myrica salicifolia* (4), *Ruta chalepensis* (29), *Vernonia amygdalina* (3), *Rumex nepalensis* (3)	23	159	0.86
Urine Retention	*Foeniculum vulgare* (6), *Rumex nepalensis* (4), *Zehneria scabra* (3)	3	13	0.83
Gynaecological disorder	*Achyranthes aspera* (4), *Solanum marginatum* (3), *Stephania abyssinica* (3)	3	10	0.78

FL is an important means to see for which ailment a particular species is more effective; and accordingly this study found ten plants (Table 10) having higher healing potential (FL > 55%) in the context of the local people to treat ailments.

**Table 10:** **FL values for some medicinal plants in Ada’a District**

**Healing plants**	**Ailments claimed to be cured**	**Tf**	**Sf**	**FL value (%)**
*Acacia albida*	Cattle eye bruise	5	5	100
*Acmella caulirhiza*	Loose tooth	7	7	100
*Gamphocarpus abyssinicus*	Blackleg	9	9	100
*Kalanchoe petitiana*	Swelling	24	24	100
*Leucas martinicensis*	General malaise	8	8	100
*Ocimum lamiifolium*	General malaise	24	24	100
*Ruta chalepensis*	Abdominal pain	29	29	100
*Leonotis raineriana*	Leech	25	15	60
*Verbena officinalis*	Tonsillitis	9	5	56
*Mysine africana*	Taeniasis	9	5	55

## Discussion and conclusion

### Medicinal plants and their occurrences in the study area

The study area yielded 131 MPs used in the traditional medical lore of the people in Ada’a District. Some of the MPs recorded from the study area were also reported by other studies to be used in the medicinal lore of other areas in Ethiopia, and it is summarized in the table below (Table 11).

**Table 11:** **Number of MP species of Ada’a District reported from studies in other parts of Ethiopia**

**Part of Ethiopia**	**Number of MP species**	**% of MP species found in Ada’a**	**Source**
Gemad, northern Ethiopia	18	13.7	[47]
Kilte Awulaelo, northern Ethiopia	52	39.6	[48]
Lake Zway Island, southern Ethiopia	15	11.4	[44]
Wonago, southern Ethiopia	43	32.8	[45]
Babile, eastern Ethiopia	9	6.8	[49]
Harla and Dengego,eastern Ethiopia	23	17.5	[33]
Assosa, western Ethiopia	14	10.6	[50]
Wayu Tuka, western Ethiopia	33	25.2	[34]
Bahirdar-zuria, Northwestern Ethiopia	10	7.6	[46]
Zegie Peninsula, Northwestern Ethiopia	27	20.6	[51]
Ada’ar, north eastern Ethiopia	5	3.8	[37]
Sekoru, southwestern Ethiopia	31	23.6	[42]
Mana Angetu, southeastern Ethiopia	27	20.6	[38]
Jeldu, west shewa	80	61.1	[32]
Ankober, north shewa	36	27.4	[35,43]

The finding of such a large number of MP species in this study area is an indication that there has been a continued transfer of plant-based traditional knowledge for generations. Moreover, highest level of shared documentation of this study was observed with [32] sharing 80 herbals in common. This is perhaps because Jeldu [32] and Ada’a district are parts of Shewa highland of Ethiopia and the people belong to the same oromo community known as Tulema where it is highly likely that they have shared norms, cultural beliefs and traditional practices.

The leading plant families that were found to encompass more numbers of medicinal plant species were the Lamiaceae, Asteraceae and Solanaceae in that order. These families are among the top plant families to contain largest medicinal plant species as reported from other parts of Ethiopia [33-35]. This could also be linked with the fact that they are reported to be among the top fifteen plant families in the flora area, Ethiopia and hence expected to contain widely distributed species in the District [36].

With regard to the growth form of medicinal plants, shrubs were found to be the widely used form followed by herbs, trees and climbers. This pattern of growth form was also reflected in other studies conducted elsewhere in Ethiopia [32,33,35,37-40]. The higher frequency of using shrubs and herbs may be due to the fact that the area is part of the wooded grassland ecosystem of the Rift Valley [41] where shrubs and herbs are leading growth forms than trees. This study also showed that most of the medicinal plants are collected from the wild environments (55.72%) and only one species, *Myrtus communis*, was recorded from open market place in the District. The wild habitats as a main occurrence site of medicinal plants are also reported in other ethnomedicinal researches conducted elsewhere in Ethiopia [33-35,38-40,42,43]. This shows that the people largely rely on wild plants; which consequently indicates the existence of higher pressure/threats on the wild medicinal plants. Thus it is a wakeup ring calling for urgent and more collaborative study to maintain the balance between their availability in the wild state and utilization by the community.

### Plant part used for remedy preparation, forms of preparation and route of provision

Among the MPs documented in this study, it is proven that majority of them are used to treat human ailments. This finding aligns with other studies elsewhere in Ethiopia [9,13,34,38,42,44,45] that reported the use of large number of medicinal plants for treating human diseases rather than domestic animals. For treating human and livestock ailments, the local people have acquainted with immense knowledge of remedy preparations.

This study also revealed that mixing of two or more MPs are common practices in remedy preparation. This is in agreement with other findings in Ethiopia [9,13,38,42] where most traditional remedies were prepared by mixing components of two or more plants. Such practices may add the healing potential and minimize the side effect it may inflict on the patient. Similar inference has also been stated in the study outputs of different ethnomedicinal researchers [9,13,33,34,38,42,44,45]. On the contrary, this current finding indicates that only few remedies were made from single plant preparations which deviate from the findings of other reports [37,46] where most of the traditional drugs in Bahirdar Zuria and Ada’ar districts respectively were made from single plant preparations. Most often, the local people of Ada’a district prefer the fresh plant part over the dried part for remedy preparation. Similar findings were reported in other areas of Ethiopia [32-35,37,38,42,44-51] and elsewhere [52-57]. This practice agrees with the scientific fact that the healing potentials of the plant are greater when fresh plant material is used for medicine preparation because the important chemicals are expected to be more and unchanged to other forms as they do when dead and dry resulting in the decline or disappearance of the active principles, usually intermediate metabolites [58].

This study reported the routes and methods of applications in the study sites varied with the type of disease treated and the position where it occurred. The most common route of applications found in this study was oral followed by dermal. This may tell us that the widespread diseases are those that are occurring internally than on the external parts of the body. Similar finding were also noted in other studies [32-35,37-40,42,43,46,50,51,53] among many others

As in most other studies, for example [33,35,43,50], roots were found to be the most familiar plant part for remedy preparations followed by leaves and fruits. Since the root is the most utilized part for remedy preparation, under intensive utilization mode it may attribute to the death of the mother plants and to the loss of the natural vegetation of the area in more severe cases. Moreover, this current finding also noted that the total above ground part (2.1%) and the whole plant (1.6%) are also parts of MPs used for remedy preparation. For example, the entire above ground part of *Eleusine floccifolia was* used to treat snake bite. Moreover, above ground part of *Foeniculum vulgare was* reported to treat urine retention. Powder made from the above ground part of *Foeniculum vulgare* was reported to treat stomach trouble. Planting whole plant of *Heteromorpha trifoliate* at the back and front yard of the house by a diviner (METSEHAF GELAC’H) was reported to save them against warding of sorcery and attack by magical thieves (SELABI). The whole plant body of *Rumex abyssinicus* was reported to treat animal scabies (EKEK). Burning the whole plant of *Artemisia abyssinica* and fumigating with the smoke was reported to treat itching eyes.

### Local diagnosis, dosages and other related prescriptions

For some diseases like stomach trouble, cough, stabbing pain and the likes local people easily diagnose, and treat them using self-prepared conventional medicines. But mostly they visit herbalists for some chronic ailments in a similar manner as reported by Kassa [32]. The also showed that the herbalists in the study area made diagnosis like that of the modern physicians that is accompanied first with case-history taking followed by physical examination. Some physical examination includes looking to the patient’s eye, or patient’s palm. Then they relate the examinations with their work experience, and infer the type of ailments and prescribe the medicine. This may at times result in wrong conclusion of ailment types and provision of local drugs that are uncalled-for.

In this study area, provision of doses varies with ages and ailment condition of the patient. Dose is not measured with standardized cylinder or balance. For instance, the root of *Achyranthes aspera* would be cut to parts simultaneously saying ‘cut the blood of so, i.e, the name of the patient’, and infused with brown tef (*Eragostis tef*), black malt and *Rhamnus prinoides* and provided to the patient to drink the infusion for three days to treat RH case (SHOTELAY); and the dose is measured by local containers (JOG or ANKOLA) and taken per day. A concoction of the root and leaf of *Justicia schimperiana* ground together with the roots of *Prunus persica*, *Nicotiana tabacum and phytolacca dodecandra* is drunk for at least a week to treat rabies. Here one tea cup per day was reported to be enough. Root and fruit of *Lagenaria siceraria* pounded together and drunk with the first boiled coffee (ABOL BUNA) using coffee cup may be enough to treat impotency (SINFET WOSIB). Leaf of *Hygrophila schulli* was reported to be powdered and the powder will be held between the thump and next (index) finger and dispersed on the wound to treat wound poisoning. Lack of consistency was also reported elsewhere in Ethiopia [33-35,44,48,49] as a serious weakness in the delivery of traditional herbals.

This study also revealed that palm sanitation of herbalists and container was not considered which could expose the drugs to contamination and thus may result in some other complications when the treatments are particularly given orally. Healers recommended not only the doses but also prescribe in how long the remedies have to be taken. Some medicines were recommended to be taken only when the patient feels the pain, or twice or three times (equivalent terms in allopathic medicine are TRN, BID and TID, respectively) in a day or days [59] and still others for a week or weeks. Some of these local drugs may be taken in the early morning before getting meal or after; some are taken early morning before urination and still others before beginning any conversation with people. Herbalists also have antidotes that are given if the herbals inflict side effects. Similar findings were reported in other studies [9,22,45,48].

### The most important MPs

Those plant species obtained through ranking by key informants have been placed in the category of priority species for any further action. *Cyphostemma adenocaule* was reported to be the most widely used plant for treating blackleg. The reasons for showing preferences may be linked with their indigenous knowledge and availability of the plants in close vicinity of the villages in the study area. Among the very common medicinal plants direct matrix showed that *Olea europaea* subsp. *cuspidata* was found to be most important in its multiple utility value similar to the findings of Lulekal *et al*. [38]. From the preference ranking it could be understood that the most favoured species is usually most efficacious at least in the context of the local people and may indicate the occurrence of bioactive chemicals responsible to ward off the causative agents, and it shall be further screened in scientific works for its pharmacological potentials.

### Degree of consensus on herbal medicines

ICF values are important guides to identify more efficacious plants; and through that way they also tell the level of prevalence of diseases in the District. This is because the traditional healing practices normally focused on the most frequent health problems. Accordingly, retained placenta, skeleto-muscular disorders, febril illness and general malaise, circulatory disorders, gastrointestinal disorders, urinary disorders, and gynaecological disorders, which turned up the highest ICF values (1.00-0.78) and hence these are the most prevalent ailment categories in the area. This finding deviates from the results obtained by Kassa [32] who found retained placenta among the least incident diseases while sudden illness and general malaise came among the most prevalent diseases. More prevalence could be linked with economic poverty and poor sanitation problems. Moreover, the dominance of retained placenta may show the lack of awareness and distribution of allopatic contraceptive methods in the District. Those plant species reported to be efficacious for the common health problems of the District are expected to be an input for pharmacologists to check the efficacy *in vitro*.

FL is an important means to see for which ailment a particular species has more healing power and accordingly those species with high FL are supposed to be more curative for the respective ailments. Thus, those traditionally used MPs with high FL can be a focus for further pharmacological tests.

### Threats of medicinal plants in the study area

Some of the MPs reported by the local people to be threatened are also included in the IUCN Red lists [60]. *Inula confertiflora*, which is under the near threatened (NT) category and *Otostegia integrifolia*, which is under the vulnerable (VU) category of the IUCN Red List are the common ones. Among those in the least concern (LC) category, *Solanecio gigas* and *Lippia adoensis* are the MPs recorded from the study area. Other MPs of Ada’a District including *Acacia abyssinica*, *Impatiens rothii*, *Jasminum stans*, *Laggera tomentosa* and *Urtica simensis* are endemic species [60-63], which are among the common threatened MPs in the study area. *Juniperus procera*, *Olea europaea* subsp. *cuspidata*, *Dodonaea angustifolia*, *Embelia schimperi were* reported to be locally threatened MPs in need of conservation efforts even if they are not under IUCN Red Lists.

## Appendix

Details on the mode of preparations and applications of remedies are given in the appendix (Table 12).

**Table 12:** **Lists of MPs collected from Ada’a District (Detail descriptions on the mode of preparations and applications)**

**S/N**	**Scientific name**	**Local name (Oromifa/Amarigna)**	**UT**	**AT (English/** ***Amaric*** **)**	**PU**	**MP A**	**IC**	**FP**	**MT**	**RA**	**CP**
1	*Acacia abyssinica* Hochst. ex Benth	Laaftoo/Girar	An	Horse scabies *(Yeferse ebitet)*	RB	Root and bark grounded together and wash the animal with the solution	3	G	W	Ex.	D
2	*Acacia albida Del*	Garbii/Gerbi	An	Eye bruise *(Bilz)*	Ba	Fresh bark masticated and spitted out on the eye	5	C	S	Ea	F
3	*Acacia seyal Del.*	Wachoo/Wachu	Hu	Headache (*Ras mitat)*	RF	Root and Fruit grounded, boil in water and breathin the smoke	3	Co	Fu	N	D
4	*Achyranthes aspera L.*	Derguu/Etse-tekeze	Hu	Stomach trouble *(Yehod hemem)*	RL	Powder of root and leaf concocted with root powder of *Allium sativum* and drunk once.	11	Co	E	Or.	D/F
			Hu	Abdominal pain in woman after birth *(Kurtet)*	L	Leaf powdered and drink the water solution	5	P	Dr.	Or.	D
			Hu	RH case *(Shotelay)*	R	Root dig out with horn handled knife wearing silver ring, cut it simultaneously saying ‘cut the blood of so and so, i. e the name of the patient’, infused with brown tef (*Eragostis tef*), black malt and *Rhamnus prinoides* and drink the infusion for three days. A glass is taken per day	4	If	Dr.	Or.	F
5	*Acmella caulirhiza Del.*	Guticha	Hu	Loose tooth	L	Leaves chewed and placed it between the aching tooth	7	C	Ho	T	F
6	*Agave sisalana* Perrine ex Engel.	Qachaa/Qacha	An	Tick	S	Stem grounded with fruit of *Solanum incanum* and polishing the bitten area	3	G	Po	Ex.	F
7	*Ageratum houstanianum Mill*	Q/Merzi/Yemerz Medanit/	Hu	Poisoning *(Merzenet)*	R	Root powder is mixed with butter and put on the affected part	5	P	Pa	Ex.	D
	*Ajuga integerifolia*	Harmmaguusa	Hu	Stomach trouble	RL	Powder of root and leaf mixed with root powder of *Allium sativum* and eaten once.	8	P	E	Or.	D/F
8	Buch. Ham	/Aqorarache/				*Allium sativum* and eaten once.					
			Hu	Cold *(Bired)*	L	Leaf boiled with tea and a cup of it is drunken	3	De	Dr.	Or.	F
			Hu	Gout *(Rihi)*	R	Root with the root of *Solanecio gigas, Leonotis rainerihana* powdered together and mixed with oil from fruit of *Olea europaea* subsp*. cuspidata* and *Datura stramonium.* Concoted them in water and drink the solution for three consecutive days. One cup only once	4	Co	Dr	Or.	D
			Hu	Hypertension *(Dem bizat)*	RL	Root and leaf boiled with tea and a cup of the solution is taken in	6	Co	Dr	Or.	F
9	*Allium cepa L.*	Shunkurtii diimaa/Key shinkurt/	Hu	Poisoning	R	Tie up the root powder with the leaf concoction of *Vernonia amygdalina, Premna schimperi*, and root powder of *Verbascum sinaiticum*	7	Co	Pa	Ex.	F
	*Allium sativum L.*	Qullubbi adii	Hu	Ascariasi	R	Root powder with the root powder of *Ajuga integerifolia, Allium sativum,* and *Rumex nepalensis* concocted together and drunk once before breakfast	5	Co	E	Or	D
10		/Nech shinkurt/		*(Wosfat)*		*integerifolia, Allium sativum,* and *Rumex nepalensis* concocted together and drunk once before breakfast					
			Hu	General malaise *(Mich)*	R	Smelling roots of a young grown plant	3		Sm	N	F
			Hu	Flu (Gunfan)	RL	Root and leaf crushed in to pieces, boiled with honey and take in the liquid in a cup of tea	8	Co	Dr	Or.	F
			Hu	Toothache *(Tirse himem)*	R	Root crushed, chewed and hold between the aching tooth	9	C	Ho	T	F
			Hu	Malaria *(Woba)*	R	Root with leaf of *Vernonia amaglidina* pounded and drunk the extracted solution	8	Pu	Dr	Or.	F
	*Aloe macrocarpa* Tod.	Argiisa/Ret	Hu	Intestinal parasite	L	Leaf chewed and swallow the juice	3	C	Sw	Or.	F
11			Bo	Swelling *(Ebach)*	L	Leaf warmed up on a fire and paste on the swelling	3	He	Pa	Ex.	F
12	*Alternanthera pungens* Kunth.	*****	Hu	Sudden illness *(Dingetegna)*	R	Fresh root boiled together with *Ocimum gratissimum* leaf and cup of the extract given to drink	3	Co	Dr	Or.	F
13	*Artemisia absinthium L.*	Harritta/Aritii	Hu	Sour throat	RL	Both root and leaf grounded together with root powder of *Verbena officinalis* and the solution sipped in	8	G	Sw	N	F
	*Artemisia abyssinica*	Tiroo/Chikugne	Hu	Whooping Cough *(Tektik)*	L	Grounded leaf boiled with the leaf powder of *Ruta chalepensis*, honey or butter and taken once a day orally	6	Co	Dr	Or.	D
			Hu	Stomach trouble	Br	Branches together with leaves of *Ajuga integerifolia* boiled with butter and eaten together with bread	12	Co	E	Or.	F
			Hu	Eye itching *(Ayenen masakek)*	WP	Burn the whole plant and fumigate with the smoke	9	Bu	Fu	Ex.	D
15	*Asparagus africanus* Lam.	Seriiti/Seriti	Hu	Amobiasis *(Ameba)*	R	Root powder mixed with honey and butter and eaten for three consecutive days before breakfast	7	P	E	Or.	D
16	*Asparagus racemosus* Wild.	Seriiti/Seriti	Hu	Amobiasis	R	Root powder mixed with honey and butter and eaten for three consecutive days before breakfast	9	P	E	Or.	D
17	*Asplenium monanthes L.*	*****	Hu	Woumb itching *(Mehatsenen masakek)*	R	Root crushed, boiled and wash the itching part with the decoction	2	De	W	Ex.	F
18	*Bersama abyssinica Fre*sen.	Loliichisa/Azamir	An	Horse Scabies *(Bech’h)*	Ba	Concoction of the bark with root and fruit of *Capparis tomentosa* is prepared. Then half of the concoction is given to drink, and the remaining half is for polish affected areas after washing	4	Co	W Dr	Ex	F
19	*Bidens pilosa L.*	Chogogitii/Chogogit	Hu	Devil sickness *(Lekefet)*	R	Root with root and leaf of *Zehneria scabra* boiled and fumigate the smoke	5	Co	Fu	Ex	F
	*Brucea antidysenterica*	Qumegno/Abalo	Hu	Evil eye *(Buda)*	R	Root powder burns in a fire and inhales the smoke	9	Bu	Fu	N	D
20	J.F. Mill		An	Colic *(yehod hemem)*	L	Leaf grounded and the water solution given to the cattle	6	G	Dr	Or.	F
21	*Buddlejia polystachya* Fresen.	Qawissa/Anfar	An	Leech *(Alekit)*	Fl	Inflorescence with the leaf of *Phytolacca dodecandra* is given in nose and ear to expel the parasite	12	Co	dr.	NE	
	*Calpurnia aurea* (Ait.)	Ceekaa/Digita	Hu	Scabies	L	Leaf boiled with leaf of *Solanecio gigas* and *stephania*	3	Co	Po	Ex.	F
22	Benth.			*(Ekek)*		*abyssinica* and drink cup of concoction twice a day.					
			An	Pubic hair louse *(Qemanjer)*	L	Grounding the leaf and wash the infected skin with the solution	3	G	W	Ex.	F
23	*Caparis tomentosa* Lam.	Goora/Gumero	Hu	Wound *(Kusil)*	R	Root powder mixed with latex of *Euphorbia tirucallii* and pasted on the wound	9	P	Pa	Ex.	E
24	*Capsicum annuum* L.	Qaara/Qariya	Hu	Amoebiasis	Fr	Fruit dried, powdered and eaten with bread baked of *Zea mays*	5	Ba	E	Or.	D
	*Carissa spinarium*	*Carissa spinarium*	Hu	Intestinal worms	R	Root grounded, dissolve in water and drunk	3	G	Dr	Or.	D
25	(Vahl.) Forssk. ex Endl.		Hu	Evil eyes	R	Root powdered and dispersed on fires to fumigate the smoke	2	Bu	Fu	Ex.	D
26	*Catha edulis* (Vahl.) Forssk. ex Endl.	Caatii/Chat	Hu	Ear infection	L	Leaf boiled with the leaf of *Croton macrostachyus* and very small juice applied once daily	3	Co	dr.	Ear	F
27	*Centella asiatica* (L.) Urban.	*****	Hu	Bleeding	R	Root powdered and paste it on the bleeding part	4	P	Pa	Ex.	D
28	*Citrus* aurantifolium (L.) Burn. f.	Loomii/Lomi	Hu	Abdominal pain *(Kurtet)*	Fr	Fruit pounded and sip the extracted liquid after meal.	5	Pu	Dr	Or.	F
29	*Clausena anisata* (Wild.) Benth.	Ulumaa/Limich	Hu	toothache	RS	Cutting root or stem, chew, place and hold on the aching tooth	7	C	Ho	Or.	F
	*Clematis simensis*	Fiitii/Enderifa	Bo	Wound	L	Leaf powdered and carefully sprinkled on the wound.	4	P	Sp	Ex.	F
30	Fresen.					It irritates if dropped on different area					
			Hu	Evil eyes	R	Root is burned and breathe in the smoke	4	Bu	In	N	D
			Hu	Wart *(Kintarot)*	L	Leaf with leaf of *Phytolacca dodecandra* powdered together and sprinkled on the wound	4	P	Sp	Ex.	F
31	*Clerodendrum myricoides* (Hochst) Vatke	Maraasisaa/misirich	Hu	Diaeehae	R	Root powder solution taken orally	4	P	Dr	Or.	D
32	*Colocasia esculenta* (L.) Schott	Godaree/Godore	Hu	Swelling	L	Leaf warmed and applied on the swelling	4	He	Pa	Ex.	F
	*Croton macrostachyus*	Bakaniisaa/Bisana	Hu	Febril illness	L	Leaf pounded, brush lips with it and kept in pocket if	6	Pu	Po	Ex.	F
33	Del.			*(Megagna)*		any one move out after meal particularly during mid sun					
			Hu	Tinea nigra *(Kuakucha)*	L	Polish an infected part with latex squeezed out from ajuvenile growing leaf	5	Ex	Po	Ex.	F
34	*Cucumis dipsaceus* Ehrenb.	Buqee seexanaa/Yesetan kil/	Hu	Depression *(Eje seb)*	L	Leaf cut either Tuesday, Thursday or Saturday early without having any conversation would be collected, pounded with leaf of *Justicia schimperiana*, and fruit of *Cucumis ficifolius*, altogether infused for three days and wash the patient with the infusion for three consecutive days	8	If	W	Ex.	F
35	*Cucumis ficifolius* A. Rich.	Holoo/Yemidir enbuay	Hu	Abdominal pain *(Kuretet)*	S	Stem chewed and sip the sap	14	C	Sw	Or.	F
36	*Cyathula cylindrica* Moq.	Derguu/Yemogn fikir	Hu	Stomachache *(Yehod hemem)*	Se	Seed milled, mixed with water and drunk the solution	4	G	Dr	Or.	D
37	*Cymbopogon citratus*	Xajisaara/Tej sar	Hu	Cough *(Sal)*	L	Blow up the leaf and inhale the smoke	4	Bu	In	Or.	F
	*Cyphostemma*	Melas golgul	An	Blackleg	R	Root pounded and boiled with root powder of	7	Co	Dr	Or.	D
38	*adenocaule (Steud. ex .A. Rich.) Descoings ex*					*Verbascum sinaiticum*, *Rumex abyssinicus* and *Rumex nepalensis* and drunk to the animal					
	Wild & Drummond		Bo	Swelling	L	Warm up the leaf and paste on the swelling	3	He	Pa	Ex.	F
			Hu	Snake bite	L	Leaf pounded and creaming all the body to avoid snake bite	4	Pu	Po	Ex.	F
39	*Datura stramonium* L.	Atsefaris/Astenagir	Hu	For Intellegency *(Letimret)*	Fr	Fruit powder mixed in a water and drunk the solution. After drinking s/he will be kept in a closed room until it is assimilated with body.	5	P	Dr	Or.	D
40	*Dodonaea angustifolia* L. f.	Etacha/Kitkita	An	Wound	L	Leaf is powdered and sprinkle on the wound	3	P	Sp	Ex.	D
41	*Dombeya torrida* (J. F. Gmel) Bamps	Daanisa/Wolkefa	Hu	Antidot for snake bites	Ba	Powder a piece of bark and sprinkle on the bitten part	3	P	Sp	Ex.	D
42	*Dregea schimperi* (Decne.) Bullock	Hida/Yeregna missa	Hu	Eczema *(Chiffea)*	Se	Seed powdered, mixed with spike powders of SENDEDO (*Pennisetum* sp.) and honey, greasing the lesion at least two times daily after washing	4	P	Po	Ex.	D
43	*Ekebergia capensis* Sparrm.	Somboo/Sombo	Hu	Syphilis *(Kitign)*	Ba	Fresh bark infused with root of *Cucumis ficifolius* for three days and the infusion drunk for a week. Amount taken per day shouldn’t go beyond the floorboard of a small coffee cup	6	If	Dr	Or.	F
	*Eleusine floccifolia*	Coqorsa/Akerma	Hu	Snake bit	Ag	Above ground part pounded and paste on the skin	3	Pu	Pa	Ex.	F
44	Forssk.		Hu	Poisoning	R	Root powder mixed with root powder of *Achyranthes aspera* and fruit of *Solanum incanum* and paste on the spot	4	P	Pa	Ex.	R
45	*Embelia schimperi* Vatke	Hanquu/Enqoqo	Hu	Tape worm *(Kosso)*	Fr	Fruit is powdered, dissolve in water, decant out the decoction and drunk early morning before meal	8	De	Dr	Or.	F
	*Eucalyptus globulus*	Nech bahir zaf	Hu	Febril illness	Br	Boiling branch and fumigate the patient with the	7	Co	Fu	Ex.	F
46	Labill.					vapour. After being fumigated the patient cased with blanket and sleep					
			Hu	Stomach trouble *(Yehod hemem)*	Br	Branch boiled, fumigate while steaming and sleep wrapping all parts of the body	4	Co	Fu	Ex.	F
47	*Euclea racemosa* Subsp*. schimperi*	Me'essaa/Dedeho	Hu	Tonsillitis *(Entil siwored)*	RL	Root and leaf together with fruit of *Hagenia abyssinica* boiled together and drink only once in a cup	5	Co	Dr	Or.	F
48	*Euphorbia abyssinica* J. F. Gmel.	Adamii/Kulkual	Hu	Haemorrhage	S	White milky sap of the plant carefully dripped on haemorrhage	6	Ex	dr.	Ex.	F
49	*Euphorbia ampliphylla*	Adamii/Kulkual	Hu	Haemorrhage	S	White milky sap of the plant carefully tapped on haemorrhage	6	Ex	dr.	Ex.	F
50	*Euphorbia tirucallii L.*	Cadaa/Kinchib	Hu	Haemorrhage	S	White milky sap of the plant carefully tapped on haemorrhage	3	Ex	dr.	Ex.	F
51	*Ferula communis L.*	Dog	Hu	cough	R	Root pulverized, burned and inhale the smoke	6	Bu	In	N	D
	*Ficus sur* Forssk.	Harbuu/Sholla	Hu	Wart on	La	Just two drops of latex from stem is applied on the	3	Ex	dr.	Ex.	F
52				hand*(Kintarot)*		wart					
			An	Swelling	L	Leaf grounded, warmed on fire and tie on the swelling	2	G	Pa	Ex.	F
	*Foeniculum vulgare*	Insilaalee/Ensilal	Hu	Urinary Retention	Ag	Above ground part is grounded, and the water solution	6	G	Dr	Or.	F
53	Mill.			*(Shinet leklekelew)*		drunk. The patient shall not take drinks and/or food for an hour.					
			Hu	Stomach trouble	Ag	Powdering the above ground part and given the solution before meal	5	P	Dr	Or.	F
	*Fuerstia africana* Th.	Eje Admek	Hu	General malaise	L	Leaf grounded and paint the patient body	10	G	Po	Ex.	F
54	Fries			(Mich)							
			An	Cattle eye disease	L	Leaf powdered, mixed with fresh butter and painting the eye for three consecutive days	13		Po	Eye	D
55	*Gamphocarpus abyssinicus* Decne.	Rebu Hunda	An	Blackleg *(Aba gorba)*	L	Same as *Prunus africana*	9	Co	Dr	Or.	D
56	*Grewia ferruginea* Hochst ex . A . Rich.	Dhoqonuu/Lenquata	Hu	Taeniasis *(Kosso)*	Ba	Fresh bark boiled together with fruit of *Hagenia abyssinica*, and the solution drunk	4	Co	Dr	Or.	F
57	*Guizotia scabra* (Vis) Chiov.	Adaa/Mech	Hu	Epilospy *(Yemitel beshita)*	R	Root powdered, boiled with root powders of *Ajuga integerifolia, Foeniculum vulgare* and *Withania somnifera*. One cup of the concoction taken orally	2	Co	Dr	Or.	D
58	*Heteromorpha trifoliata* (Wendel. ) Eckl. & Zeyh.	Demehee/Yejib merk uze	Hu	Warding of Sorcery Stealing *(Selabi)*	Wp	Whole plant planted at the back of the house and on front yard by a diviner (Metsehaf Gelac’h)	5			-	-
59	*Hygrophila schulli* (Hamilt.) M. R. & S. M. Almeida	Q/Mearzi	Bo	poisoning	L	Leaf powdered and dispersed on the wound	3	P	Sp	Ex.	D
60	*Hypericum quartinianum* A. Rich.	Muke fonii	Hu	Jaundice *(Yewof beshita)*	L	Leaf with roots of *Asparagus sp*. pounded and homogenised in water and given to the patient orally for three consecutive days. Half a glass is the limit for a day	4	Co	Dr	Or.	D
61	*Hypericum revolutum* Vahl	Hindhee/Ameja	An	Eye disease	R	Root with leaf of *Inula confertiflora* chewed and spitted on the eye	3	C	S	Ey	F
62	*Impatiens ethiopica* Grey-Wilson	Yehenshoshilaa zer	Hu	Wound	R	Root pounded, warmed in a dish on a fire, and creaming palms	2	Pu	Po	Ex.	F
63	*Impatiens rothii* Hook. f.	Buri/Gesherit	Hu	Wounds on hand	R	Root pounded in to pieces and thoroughly warmed on fire and firmly hands them for drying the wound	2	Pu	Po	Ex	F
64	*Impatiens tinctoria* A. Rich. Subsp. *abyssinica* (Hook. f.) Grey-Wilson	Ensosilla	Hu	Wound on palm	R	Root pounded, warmed in a dish on a fire, and oiling palms	2	Pu	Po	Ex.	F
	*Inula confertiflora* A.	Mognoree/Weynageft	An	Eye disease	R	Root with *Hypericum revolutum* chewed together and	3	C	S	Ey	F
65	Rich					spitted on the eye					
			B	Rabies *(Yehebid wusha beshita)*	R	Root with root *of Lagenaria siceraria, Stephania*	2	Co	Po	Or.	D
						*abyssinica, Verbascum sinaiticum* and young growing leaves of *Laggera tomentosa, Croton macrostachyus* with fruit of *Solanum anguivi* all together grounded and boiled, and drink with milk; or the concoction creamed on meat and eaten					
	*Jasminum grandiflorum*	Qamaxee/Tembelel	Hu	Evil eye	R	Root burned and draw in the smoke	3	Bu	In	N	D
66	L.		Hu	Toothache *(Yeters himem)*	S	Young stem chewed and hold between the aching tooth	3	C	Ho	T	F
67	*Juniperus procera* Endle	Gaatiraa/Yehabesha Tid	Hu	Demon possesesion *(Ganen)*	Fr	Fruit powder boiled with root of *phytolacca dodecandra and fruit powder of Datura stramonium;* and wash the patient for three days	4	Co	Dr	Or.	D
	*Justicia schimperiana*	Dhumuugaa/Sensel	Hu	Jaundice	L	Newly growing leaves of seven different individual	4	Pu	Dr	Or.	F
68	(Hochst. ex Nees) T. Anders			(Gubet beshita)		plants milled on palms and the squeezed liquid added to a coffee cup. Drink the liquid every morning for a week. Antidotes recommended is to eat porridge of black teff (*Eragostis tef*) and drinking local beer (tella)					
			An	Sheep diarrhoea	L	Leaf grounded and mixed with half cup water. The solution is then drunk	9	G	Dr	Or.	F
			An	Blackleg	L	Leaf powder boiled with root powder of *Rubia cordifolia* and bark powder of *Prunus africana* and given to the cattle	8	Co	Dr	Or.	D
			Hu	Rabies	LR	Root and leaf boiled together with root of *Prunus persica, Nicotiana tabacum and phytolacca dodecandra* and drink the solution for at least a week. One tea cup per day is the limit	6	Co	Dr	Or.	D
69	*Kalanchoe petitiana* A. Rich	Bosoqee/Endahula	Bo	Swelling	L	Leaf warmed up on a fire and paste on the swelling	24	He	Pa	Ex.	F
70	*Lagenaria siceraria*	Buqqee/Kil	Hu	Impotency	RF	Root and fruit grounded together and drink with the	3	G	Dr	Or.	D
	(Molina) Standl			*(Sinfet wosib)*		first boiled coffee (ABOL BUNA)					
71	*Laggera tomentosa* (Sch. Bip. ex A. Rich.) Oliv. & Hiern	Keskeso	Hu	Flu (Gunfan)	L	Actively growing leaves collected and pounded together, wrapped in a piece of cloth and place in noses	3	Pu	Is	N	F
72	*Lantana camara L.*	Yewof kolo	Hu	General malaise *(Mich)*	L	Leaf pounded together with leaf of *Ocimum lamiifolium* and the squeezed out liquid drink with coffee	2	Pu	Dr	Or.	F
73	*Leonotis raineriana* Vis.	Bokkoluu dimma /Ras kimir/	An	Leech	LF	Leaves and flowers grounded and the water solution given to the animal to expel out the parasites	15	G	Dr	Or.	D
			Hu	General malaise *(Mich)*	L	Leaf pounded together with leaf of *Ocimum lamiifolium* and the squeezed out liquid drink with coffee	10	Pu	Dr	Or.	F
74	*Leucas martinicensis* (Jacq. ) R. Br.	Bokkoluu adii/Ras kimir	Hu	General malaise *(Mich)*	L	Leaf pounded together with leaf of *Ocimum lamiifolium* and the squeezed out liquid drink with coffee	8	Pu	Dr	Or.	F
75	*Lippia adoensis* Hochst. ex Walp.	Kusaayee/Kese	Hu	Kusaayee /Kese	L	Leaf grounded, boiled in water with root powder of *Stephania abyssinica* and given to the patient	3	Co	Dr	Or.	D
76	*Maesa lanceolata* Forssk.	Abbayyii/Kelewa	Bo	Swelling	L	Leaf warmed up on a fire and paste on the swelling	5	He	Pa	Ex.	F
77	*Malva venticillata L.*	Liitii/Lit	An	Swelling	L	Leaf crushed, warmed on fire and tie on the swelling	2	He	Pa	Ex.	F
	*Melia azedarach L.*	Melia	An	Chicken diarrhae	L	Leaf grounded mixed with INJERA and given to the	3	G	E	Or.	D
78						chicken					
			An	Cattle diarrhae	L	Leaf infused in a litter of water; and the infusion is given for the cattle	3	If	Dr	Or.	F
			Hu	Taeniasis *(Kosso)*	L	Leaf grounded with water and the solution given to the patient orally	3	G	Dr	Or.	D
79	*Mentha spicata*	Nana	Hu	Cough and cold	L	Leaf boiled with tea and drunk	4	De	Dr	Or.	F
80	*Myrica salicifolia* A. Rich.	Kataba/Shinet	Hu	Ascariasis	R	Water solution of the root infusion is given orally	4	If	Dr	Or.	D
	*Myrsine africana L.*	Qacama/Kechem	Hu	Taeniasis	Fr	Fruit grounded and concocted with powder fruit of	5	Co	Dr	Or.	D
81						*Hagenia abyssinica*; and the solution drunk					
			An	Worms in donkey	Fr	Decocting fruit with local beer (TELLA) and drink the solution	4	De	Dr	Or.	D
82	*Myrtus communis*	Adasii/Ades	Hu	General malaise	L	Leaf burned and fumigated to the smoke	3	Bu	Fu	Ex.	D
			An	Colic	L	Leaf grounded with fruits of *Embelia schimperi* and	3	Co	Dr	Or.	D
						leaf of *Vernonia amygdalina* and drunk the cattle					
83	*Nicotiana tabacum L.*	Tamboo/Tembaho	Hu	Exhausion *(Lib dikam)*	Fr	Fruit grounded and boiled with root powder of *Asparagus africanus* and *Cynoglossum geometricum* and take in the solution	3	Co	Dr	Or.	D
84	*Ocimum basilicum L.*	Besobilla	Hu	Flu	L	Leaf together with fruit of *capsicum annuum* and root of *Aloe macrocarpa* concocted together and drink the solution	5	Co	Dr	Or.	F
85	*Ocimum gratissimum* L	Q/Michii/Mech medanit	Hu	General malaise	L	Leaf rubbed between palms & drink the juice	15	Pu	Dr	Or.	F
86	*Ocimum lamiifolium* Hochst. ex Benth.	Demakessie	Hu	General malaise	L	Leaf pounded with leaf of *Lippia adoensis* and *Fuerstia africana* .The squeezed out solution drink with ABOL BUNA (first boiled coffee) and body will be creamed with the remaining leaves	24	Pu	Dr	Or.	F
87	*Olea europaea* L. subsp. *cuspidata* (Wall. ex G. Don) Cif.	Ejersa/Weyra	Hu	*QOROQOR*	La	Latex or oil of any part of the plant is greased on the head for a week	4	Ex	Po	Ex.	F
	*Olinia rochetiana* A.	Dalecho	Hu	Snake bite	La	Latex from any part of the plant preferably from root	2	Ex	Pa	Ex.	F
88	Juss.					mixed with Leaf powder *Ficus Vasta* and pasted on the wound					
			Hu	Toothache	L	Young growing leaves cut from three different individual plants, chewed them and hold between the aching tooth	4	C	Ho	T	F
89	*Osyris quadripartita* Decn.	Waatoo/Qeret	Hu	Eczema	La	Latex is mixed with spikes of SINDEDO (*Pinnesetum* sp.) and creaming the affected areas	3	Ex	Po	Ex.	F
90	*Otostegia integrifolia* Benth.	Tungiitii/Tungit	Hu	Fibril illness *(Megagna)*	L	Smoke the leaf and fumigate the house for a woman who deliver baby	12	Bu	Fu	Ex.	F
	*Pavetta abyssinica*	Muke-buniti	Hu	Poison	R	Root boiled with leaves of *Bersama abyssinica* and	2	Co	W	Ex.	F
91	Fresen.					*Rumex abyssinicus* and wash the wound with the solution					
			An	Animal diarrhoea	L	Ground the leaf and drunk the solution	3	G	Dr	Or.	D
92	*Pentas schimperiana* (A. Rich.) Vatke	Dasie	An	Eye disease	S	Stem is chewed and spitted on the eye	11	C	S	E	F
	*Phytolacca dodecandra*	Handoode/Endod	An	*BECHE’H*	L	Leaf grounded and wash the area with the solution	10	G	W	Ex.	D
93	L’ Herit		Hu	Wart on hand	L	Leaf together with *Physalis peruviana* dried , grounded and drip a drop or two on the wart	9	Co	dr.	Ex.	D
94	*Plantago lanceolata L.*	Qorxobbii/Yehaheya Kote/	An	Intestinal parasite	L	Leaf grounded, boiled with fruit of *Solanum anguivi* and the concoction mixed with ATELA (by-products of TELLA) and given to the animal	4	Co	Dr	Or.	F
	*Plantago major L.*	Qorxobbii	Hu	Poisoning	R	Root powder is employed to bandage wounds of any	3	P	Pa	Ex.	D
95		/Yekura wesife/				kind					
			Hu	Haemorroides	L	Leaf grounded and place it on the wound	3	G	Pa	Ex.	D
96	*Premna schimperi* Engl.	Urgessa/Chchoho	Hu	Eye disease	L	Leaf pounded with leaf of *Buddlejia polystachya*, and	4	Pu	dr.	Ey	F
						the juice is dripped on the eye					
97	*Protea gaguedi* J. F.	Dasie	An	Animal jaundice	L	Fresh leaf grounded, infused for a day and the solution	11	If	Dr	Or.	F
	Gmel.					given to the animal					
	*Prunus africana* (Hook.	Hoomii/Tikur Enchet	Hu	Swelling	Ba	Bark dried well, grounded, boiled with water, filtered	4	De	Dr	Or.	D
98	f. ) Kalms					and drunk					
			Hu	Sudden illness *(Dingetegna)*	Ba	Bark dried well, grounded, boiled, filtered and drunk	6	De	Dr	Or.	D
			An	Blackleg	Ba	Bark powdered, boiled with leaf powder of *Gamphocarpus abyssinicus* and the solution given to the animal	2	Co	Dr	Or.	D
			An	Anthrax *(Abasenga)*	Ba	Bark powdered, mixed with leaf powder of *Gamphocarpus abyssinicus* and the solution given to the animal	2	P	Dr	Or.	D
99	*Prunus persica* (L.) Batsch	Kokii/Kok	Hu	Epistaxis *(Neser)*	R	Root chewed and band in cloth and place in nose	2	C	Is	N	F
100	*Pterolobium stellatum* (Forssk. ) Brenan	Harengeemmaa/Kontir	Hu	Rhumantic pain *(Kurtimat)*	R	Root boiled in a cooking dish and fumigating the leg with vapour.	5	De	Fu	Ex.	F
101	*Rhus retinorrhoea*	Tilem	An	Anthrax *(Aba senga)*	Ba	The bark is grounded, boiled with leaf powder of *Phytolacca dodecandra* in a cup of water. Drink the solution to the animal for a week	4	Co	Dr	Or.	F
102	*Rhus vulgaris* Meikle	Dabobechaa/Kimmo	An	Diarrhoea	L	The leaf together with the leaf of *Premna schimperi* and *Clerodendrum myricoides* is concocted together. The concoction is given to the cattle	3	Co	Dr	Or.	F
103	*Ricinus communis L.*	Qoboo/Gulo	Hu	Dandruff *(Forofor)*	FL	Fruit and leaf pounded together and paint the patient’s head skin	6	Pu	Po	Ex.	D
104	*Rosa abyssinica* Lindley	Gora/Kega	An	Invoking sprit *(Aganent)*	L	Leaf infusion together with leaf powder of *Vernonia amygdalina* is prepared and small amount of the solution placed through nose and the remaining solution is for painting the body	7	If	Po	N	F
105	*Rosmarinus officinalis L.*	Siga metsebesha	Hu	Headache	R	Root powder mixed powder root of *Ocimum gratissimum* and drunk the solution	13	P	Dr	Or.	D
	*Rubia cordifolia L.*	Enchiberii/Enchibir	Hu	Wound	R	Root grounded and sprinkle the powder on the wound	13	P	Sp	Ex.	D
106			Hu	Cough	R	Root is grounded and drink with tea	6	G	Dr	Or.	D
			Hu	Cough	R	Root grounded, homogenised and drunk with butter stayed for 7 years. One glass is the limit for adult and a cup is for children	7	G	Dr	Or.	D
			An	Cataract *(Bemora yete-shefene ayen)*	R	Root chewed with root of *Fuerstia africana* and spitted on the cattle’s eye	5	C	S	Ey	F
	*Rubus steudneri*	Agogota	Hu	Stabbing pain	L	Leaf powdered, mixed with EMAMESA QEBE (	2	P	Po	Ex.	D
107	Schweinf.			*(Wugat)*		butter from a cow with same colour calf), and cream it on the area butter from a cow with same colour calf), and cream it on the area					
			Hu	Cough	L	Leaf with root of *Rubia cordifolia* and Leaf of *Thymus schimperi* boil together with butter and drink	2	Co	Dr	Or.	F
	*Rumex abyssinicus* Jacq.	Meqmeqo	Hu	Eye bruise	R	Root washed, crushed and boiled with butter. One	5	Co	Dr	Or.	F
108						glass of the solution drunk per day					
			An	Blackleg	R	Root powdered and given to the cattle with ATELLA (By product of TELLA)	2	P	Dr	Or.	D
			An	Scabies *(Ekek)*	WP	Whole plant pounded, mixed with water and wash the animal with it	2	Pu	W	Ex.	F
	*Rumex nepalensis*	Shuultii/Tulet	An	Colic *(Yehod himem)*	R	Root grounded and drink the water solution	3	G	Dr	Or	D
109	Spreng.		An	Blackleg	R	Root with root of *Clematis simensis* and *Rubia cordifolia* boiled and drink the animal	6	co	Dr	Or.	D
			Hu	Stomach pain *(Cheguara)*	R	Root powdered, disperse it in water and drink the solution	3	P	Dr	Or.	D
			Hu	Stabbing pain *(Wugat)*	R	Root grounded and drink the solution once in a tea cup	3	G	Dr	Or.	D
			B	Urinary retention	R	Root with leaf of *Foeniculum vulgare* boiled together and drink	4	Co	Dr	Or.	F
110	*Rumex nervosus* Vahl	Dhangaggoo/Embuacho/	Hu	Delay in drying circumcision	R	Root pounded and paste on the penis forehead	5	Pu	Pa	Ex.	F
111	*Ruta chalepensis L.*	Xeenaadama/Tsenadam/	Hu	Abdominal pain	R	Root chewed and ingest the juice	29	C	Sw	Or.	F
112	*Salix mucronata*	Alaletu/Ahaya	Hu	*MIKEGNA-SHEREGNA*	L	Chewing the leaf and sipping it in	6	C	Sw	Or.	F
113	*Salvia nilotica* Jacq	Hulegebe	Hu	Wound	R	Root powder mixed with butter and applied to wound	3	P	Pa	Ex.	D
114	*Schinus molle L.*	Kundoberbere zaf	Hu	Wound on rectal area	R	Root powder mixed with resin of *Euclea racemosa* Subsp*. schimperi* and applied on the wound once a day	3	P	Pa	Ex.	F
	*Sida schimperiana*	Chefreg	An	Rabies	R	Root dried, powdered, and baked with flour of Tikur	2	Ba	E	Or.	D
115	Hochst. ex A. Rich.					teff (*Eragostis tef*) and given to eat against rabies before an animal bitten by a mud dog	2	Ba	E	Or.	D
			An	Preventing bitch birth	L	Leaves infusion mixed with fruit of *Solanum incanum* is prepared and drunk	2	If	Dr	Or.	D
116	*Snowdenia polystachya* (Fresen.) Pig.	Muja	Hu	Scabies *(Ekek)*	R	Root boiled with root of *Carissa spinarium* and wash the animal with the concoction	2	Co	W	Ex.	D
117	*Solanecio gigas* (Vatke.)	Gommana osolee	Bo	Retained placenta	L	Grounding the leaf and drinking the solution. In any	7	G	Dr	Or.	D
	C. Jeffrey	/Yeshikoko gomen/				case the dose shall not exceed a cup					
	*Solanum anguivi* Lam.	Hiddi Worabessa	Hu	Intelligence	Se	Seed grounded, boiled with leaf of *Datura*	3	Co	Dr	Or.	F
118		/Zerch Enbuay/				*stramonium* and small amount given to a child to be a fast learner					
			Hu	Danruff	Fr	Fruit together with leaf of *Acacia albida* and *Ruta chalepensis* powdered, mixed with Vaseline and paint the head	2	P	Po	Ex.	D
			An	Rabies	Fr	Fruit powder baked with brown teff (*Eragostis tef*) and given to dog against rabies. This is given to healthier dog not to be infected with rabies	3	Ba	E	Or.	D
	*Solanum incanum L.*	Hiddii	An	Tick bite	Fr	Infusion of fresh fruit mixed with kerosene gas and	2	If	Sr	Ex.	F
119		/Yehabesha Embuay/				spray on the skin of the animal					
			An	Horse Scabies	R	Pounding the root with carbon (carbon rod of dry cell) and spraying the water solution on the infected part.	2	Pu	Sr	Ex.	F
			Hu	Wounds	L/Fr	Pounding the fresh leaf and fruit and drip a drop of the extract on the wound	2	Pu	dr.	Ex.	F
120	*Solanum marginatum* Linn. f.	Hiddii/Tileku Enbuay	Hu	Long stay menstruation	R	Root powdered with roots powder of *Achyranthes aspera, Solanum incanum, Jasminum grandiflorum,* wrapped together, woven three times over head and between legs and tied around the waist	5	P	Tw	Ex.	D
	*Stephania abyssinica*	Kalaala/Engochit	An	Rabies	R	Root powder baked with flour of brown tef (*Eragostis*	6	Ba	E	Or	D
121	(Dillon ex A. Rich.)					tef) and small amount given once.					
	Walp.		An	Blackleg	R	Root powders of the plant and *Rumex abyssinicus*,*Cyphostemma adenocaule*, *Eucalyptus globules*, *Allium sativum*, and *Solanum incanum* mixed together in a litter of water and drink once	5	co	Dr	Or.	D
			Hu	Unwanted pregenancy	R	15-20 cm fresh root whose upper part chewed inserted to the womb for aborting unwanted pregnancy	3	C	Is	V	F
			Hu	Wound	R	Either powdered or fresh root boiled with leaf of *smilex aspera* and wash the wound	3	Co	Pa	Ex.	D/F
			Hu	Swelling	R	Root grounded and dissolved in a cup of water and drink. The recommended antidotes is to drink ABOL BUNA with no sugar	5	P	Dr	Or.	P
			Hu	Sudden illness	R	Little sized root chewed and only small amount of the extract swallowed	4	C	Sw	Or.	F
122	*Tagetes minuta L.*	Tiro	An	*KINKIN*	Ag	Above ground part collected from field and sleep chickens on it	4			Ex.	F
123	*Thunbergia alata* Sims.	Hareg	Hu	Cough	R	Powder of root taken once mixed with honey	3	P	E	Or.	D
124	*Thymus schimperi* Ronniger	Xoosanyii/Tosigne	Hu	Hypertension	R	Root dried, powdered, and drink with tea	8	P	Dr	Or.	D
125	*Urtica simensis* Steudel	Dobii/Sama	Hu	Gonorrheae (Chebit)	R	Infusion of Root is prepared and the genital organ washes with it once daily.	2	If	W	Ex.	F
	*Verbascum sinaiticum*	Guraa Haree	Hu	Nightmare	R	Root crashed, placed in a fire and fumigating the	4	Bu	Fu	Ex.	D
126	Benth.	/Yahaya joro/				smoke					
			An	Blackleg	R	Root with *Phytolacca dodecandra* leaf boiled and the solution drunk	2	Co	Dr	Or.	D
	*Verbena officinalis L.q*	Atuch	Hu	Cough	R	Powdered root with the root of *Rubia cordifolia*	4	Co	E	Or.	D
127						cooked with butter; and the concoction taken once daily					
			Hu	Tonsilities *(Entil siwored)*	R	Root is chewed and ingesting the juice	5	C	Sw	Or.	F
	*Vernonia amygdalina*	Ebicha/Grawa	Hu	Warding off sorcery steeling	L	Infusion of leaf powder kept for a night and will be sprayed on fences early of the following morning	5	If	Sr		D
			Hu	Malaria	F	Leaf is grounded and the solution taken orally	5	G	Dr	Or.	D
			Hu	Abdominal pain	L	Leaf is grounded and the solution taken orally	3	G	Dr	Or.	D
129	*Withania somnifera* (L.) Dunal.	Gizaawaa/Gizawa	Hu	Daemon possesesion	R	Root powder mix with root powder of KEBERICHO (*Echinops kebericho)*and smoking in a house who delivered baby	6	P	Bu		D
130	*Xanthium strumarium L.*	Yemogne Fikir	An	Leech	L	Leaf juice of *Xanthium. strumrium, Clematis simensis, Calpurnia aurea* applied through nose and ear of the animal to expel the parasite	3	Pu	dr.	NE	F
131	*Zehneria scabra L.*	Daaymii/Areg resa	Hu	Deformed lips *(Megagna)*	R	Water solution of the grounded root drunk and some paint near the abnormally deformed lips	6	G	Dr	Or.	D
			Hu	Urinary retention	R	Root powdered and drink the water solution	3	P	Dr	Or.	D

Key: **UT**-used to treat (An=domestic animal, Hu=Human, Bo=both), **AT**-Ailments treated, **PU**-Part used (Ag=above ground part, Ba=bark, Br=branch, Fl=flowers or inflorescence, Fr=fruit, L=leaf, L/fr=leaf and fruit, La=latex or resin, Lf=leaf and inflorescence, R=root, RB=root and bark, RF=root and fruit, RL=root and leaf, RS=root and stem, S=stem, Se=Seed, WP=whole plant), **IC**- total number of informants who cited the MPs for treating the major aliments **FP**-forms of preparation (Ba=baking, Bu=burning, C=chewing, Co=concoction, De=decoction, G=grinding, Ex= Exudate collection He=heating or warming, If=infusion, P=powdering, Pu=pounding, Wp=without processing), **MT**-means of treatment (Bu=smoking, Dr=drinking, dr.=dripping, E=eating, Fu=fumigating, Ho=holding between aching tooth, In=inhaling, Is=inserting, Pa=pasting, Po=polishing or creaming, S=spitting on eye, Sm=smelling, Sp=sprinkling, Sr=spraying, Sw=swallowing chewed juice, Tw=tie around waist, W=washing), **RA**-routs of application (Ea=ear or auricular, Ex=external or dermal, Ey=Eye or ocular, N=nasal=NE=nasal and through ear, Or=oral, T=on tooth, V=vaginal **CP**-condition of preparation (D=dry, F=fresh, DF=dry or fresh). *****- Local name not known.
